# Full Genome Characterization of Novel DS-1-Like G8P[8] Rotavirus Strains that Have Emerged in Thailand: Reassortment of Bovine and Human Rotavirus Gene Segments in Emerging DS-1-Like Intergenogroup Reassortant Strains

**DOI:** 10.1371/journal.pone.0165826

**Published:** 2016-11-01

**Authors:** Ratana Tacharoenmuang, Satoshi Komoto, Ratigorn Guntapong, Tomihiko Ide, Phakapun Sinchai, Sompong Upachai, Tetsushi Yoshikawa, Piyanit Tharmaphornpilas, Somchai Sangkitporn, Koki Taniguchi

**Affiliations:** 1 National Institute of Health, Department of Medical Sciences, Nonthaburi, Thailand; 2 Department of Virology and Parasitology, Fujita Health University School of Medicine, Toyoake, Aichi, Japan; 3 Department of Pediatrics, Fujita Health University School of Medicine, Toyoake, Aichi, Japan; 4 Department of Disease Control, Ministry of Public Health, Nonthaburi, Thailand; University of Hong Kong, HONG KONG

## Abstract

The emergence and rapid spread of unusual DS-1-like intergenogroup reassortant rotavirus strains have been recently reported in Asia, Australia, and Europe. During rotavirus surveillance in Thailand in 2013–2014, novel DS-1-like intergenogroup reassortant strains having G8P[8] genotypes (i.e., strains KKL-17, PCB-79, PCB-84, PCB-85, PCB-103, SKT-107, SWL-12, NP-130, PCB-656, SKT-457, SSKT-269, and SSL-55) were identified in stool samples from hospitalized children with severe diarrhea. In this study, we determined and characterized the complete genomes of these 12 strains (seven strains, KKL-17, PCB-79, PCB-84, PCB-85, PCB-103, SKT-107, and SWL-12, found in 2013 (2013 strains), and five, NP-130, PCB-656, SKT-457, SSKT-269, and SSL-55, in 2014 (2014 strains)). On full genomic analysis, all 12 strains showed a unique genotype constellation comprising a mixture of genogroup 1 and 2 genes: G8-P[8]-I2-R2-C2-M2-A2-N2-T2-E2-H2. With the exception of the G genotype, the unique genotype constellation of the 12 strains (P[8]-I2-R2-C2-M2-A2-N2-T2-E2-H2) was found to be shared with DS-1-like intergenogroup reassortant strains. On phylogenetic analysis, six of the 11 genes of the 2013 strains (VP4, VP2, VP3, NSP1, NSP3, and NSP5) appeared to have originated from DS-1-like intergenogroup reassortant strains, while the remaining four (VP7, VP6, VP1, and NSP2) and one (NSP4) gene appeared to be of bovine and human origin, respectively. Thus, the 2013 strains appeared to be reassortant strains as to DS-1-like intergenogroup reassortant, bovine, bovine-like human, and/or human rotaviruses. On the other hand, five of the 11 genes of the 2014 strains (VP4, VP2, VP3, NSP1, and NSP3) appeared to have originated from DS-1-like intergenogroup reassortant strains, while three (VP7, VP1, and NSP2) and one (NSP4) were assumed to be of bovine and human origin, respectively. Notably, the remaining two genes, VP6 and NSP5, of the 2014 strains appeared to have originated from locally circulating DS-1-like G2P[4] human rotaviruses. Thus, the 2014 strains were assumed to be multiple reassortment strains as to DS-1-like intergenogroup reassortant, bovine, bovine-like human, human, and/or locally circulating DS-1-like G2P[4] human rotaviruses. Overall, the great genomic diversity among the DS-1-like intergenogroup reassortant strains seemed to have been generated through additional reassortment events involving animal and human strains. Moreover, all the 11 genes of three of the 2014 strains, NP-130, PCB-656, and SSL-55, were very closely related to those of Vietnamese DS-1-like G8P[8] strains that emerged in 2014–2015, indicating the derivation of these DS-1-like G8P[8] strains from a common ancestor. To our knowledge, this is the first report on full genome-based characterization of DS-1-like G8P[8] strains that have emerged in Thailand. Our observations will add to our growing understanding of the evolutionary patterns of emerging DS-1-like intergenogroup reassortant strains.

## Introduction

Group A rotaviruses (RVAs), members of the *Reoviridae* family, are the leading pathogens causing diarrhea in young children and many animal species worldwide. In humans, RVA disease is associated with high morbidity and mortality, being responsible for an estimated 453,000 deaths among children <5 years of age annually [1], with more than half of the deaths occurring in developing countries in Asia and Africa [1, 2]. The RVA virion is a triple-layered, non-enveloped icosahedron with an 11-segment genome of double-stranded (ds)RNA, encoding six structural proteins (VP1-4, VP6, and VP7) and six non-structural proteins (NSP1-6) [3]. The segmented nature of the genome facilitates reassortment between/within human and animal strains, and the reassortment plays one of the major roles in the generation of the genomic diversity of RVAs [4].

The RVA virion has two outer capsid proteins, VP7 and VP4, which are independently involved in viral neutralization, and define the G and P genotypes, respectively. Thus far, RVAs have been classified into at least 27 G and 37 P genotypes [5, 6]. Although several G/P genotype combinations have been reported, some specific G and P genotypes are commonly found in individual host species [7, 8]. Regarding human RVAs (HuRVAs), G genotypes G1-4, G9, and G12, and P genotypes P[4], P[6], and P[8] are considered as major genotypes [9, 10]. In addition, several G (G5, G6, G8, G10, G11, and G20) and P (P[1]-[3], P[5], P[7], P[9]-[11], P[14], P[19], and P[25]) genotypes have been detected sporadically in humans [11–14]. Many of these uncommon genotypes are believed to have originated from animal RVAs that were introduced into the human population through interspecies transmission events [7, 8, 15, 16].

A full genome-based genotyping system was recently established for RVAs based on the assignment to all the 11 gene segments to provide a better understanding of the overall genetic relatedness among RVA strains (i.e., G/P and non-G/P defining genes) [17]. In the new genotyping system, the nomenclature Gx-P[x]-Ix-Rx-Cx-Mx-Ax-Nx-Tx-Ex-Hx, where x is an integer, defines the genotypes of the VP7-VP4-VP6-VP1-VP2-VP3-NSP1-NSP2-NSP3-NSP4-NSP5 genes of a given RVA strain. Most HuRVAs have gene segments similar in sequence to those of prototype human strain Wa (genogroup 1 genes) or DS-1 (genogroup 2 genes) [18]. The Wa-like strains are characterized by non-G/P genotypes comprising I1-R1-C1-M1-A1-N1-T1-E1-H1, and tend to possess G/P genotypes G1P[8], G3P[8], G4P[8], G9P[8], G12P[6], and G12P[8] [19]. In contrast, the DS-1-like strains are characterized by non-G/P genotypes comprising I2-R2-C2-M2-A2-N2-T2-E2-H2, and tend to possess G/P genotype G2P[4]. Genogroup 1 and 2 genes of HuRVAs are believed to have originated from distinct animal species [17]. Although intergenogroup reassortant strains can be generated, it is believed that such RVA strains possessing both genogroup 1 and 2 genes may have decreased evolutionary fitness compared to that of the parental strains and thus would be selected against in the context of human infections [18–20]. However, the emergence of novel human intergenogroup reassortant strains, DS-1-like G1P[8] strains and reassortant ones derived from them possessing a DS-1-like non-G/P genotype constellation (I2-R2-C2-M2-A2-N2-T2-E2-H2) together with G/P genotypes G1P[8], G2P[8], G3P[8], and G8P[8]: G1/2/3/8-P[8]-I2-R2-C2-M2-A2-N2-T2-E2-H2, were recently reported in Japan, Thailand, Vietnam, Australia, Hungary, and Spain [19, 21–27].

The first DS-1-like intergenogroup reassortant strains, DS-1-like G1P[8] strains (including strains HC12016, NT004, and OH3506), were detected in diarrheic children in Japan in 2012 [24, 27, 28], and subsequently, these unusual strains (including strain SKT-109) were identified in Thailand in 2013 [26]. In 2013, DS-1-like intergenogroup reassortant strains having G2P[8] and G3P[8] genotypes were also detected in Thailand (including strains LS-04 (G2P[8]) and SKT-281 (G3P[8])) and Australia (including strain D388 (G3P[8])) [19, 22]. Furthermore, in 2013–2014, we identified DS-1-like intergenogroup reassortant strains having G8P[8] genotypes (including strains KKL-17, PCB-79, PCB-84, PCB-85, PCB-103, SKT-107, SWL-12, NP-130, PCB-656, SKT-457, SSKT-269, and SSL-55) in Thailand as major strains [29]. Similar DS-1-like G8P[8] strains (including strain RVN1149) were subsequently detected in Vietnam in 2014–2015 [25]. G8, like G6 and G10, is one of the common G genotypes in cattle that has been identified in humans sporadically [30]. Although human G8 strains are particularly prevalent in Africa, they have been infrequently detected elsewhere [25, 31]. In 2015, the emergence of DS-1-like G3P[8] strains (including strains ERN8263 and SS96217158) was recognized in Hungary and Spain, Europe [21, 23].

Full genome-based analysis is a reliable method for obtaining precise information on the origin of an RVA strain, and for tracing its evolutionary dynamics [15, 17, 19, 27]. To date, however, the full genome sequences of only two DS-1-like intergenogroup reassortant strains having G8P[8] genotypes, strain RVN1149 from Vietnam and strain DRC88 from the Democratic Republic of Congo, have been sequenced and characterized, indicating the presence of bovine-like segments on a DS-1-like genetic backbone [25, 32]. However, because the overall genomic constellation and genomic diversity of DS-1-like G8P[8] strains remain to be elucidated, full genomic analysis of Thai DS-1-like G8P[8] strains might be useful for obtaining a more precise understanding of the evolutionary dynamics of the emerging DS-1-like intergenogroup reassortant strains involving DS-1-like G8P[8] HuRVAs. In the present study, we analyzed the full genomes of 12 DS-1-like G8P[8] strains that have emerged in Thailand. Furthermore, the full genomes of two locally circulating strains (DS-1-like G2P[4] HuRVAs) were also sequenced as references.

## Materials and Methods

### Ethics Statement

The study was approved by the Ethical Review Committee for Research on Human Subjects of the Ministry of Public Health, Thailand (Ref. no. 10/2555). In this study, written informed consent for the testing of stool specimens for rotaviruses and characterization of detected RVA strains was obtained from the children’s parents/guardians.

### Virus Strains

The full-genomic sequences were determined for strains KKL-17, PCB-79, PCB-84, PCB-85, PCB-103, SKT-107, SWL-12, NP-130, PCB-656, SKT-457, SSKT-269, and SSL-55, and locally circulating strains LS-202 and LS-L7, which were detected in 14 stool specimens from hospitalized children aged 11–54 months with severe gastroenteritis in the Phechaboon and Sukhothai Provinces, Thailand, during the RVA surveillance program in those provinces in 2013–2014, which involved 666 RVA-positive fecal samples and 133 G8P[8] strains with a short electropherotype [29]. Out of the 133 G8P[8] strains, a representative 12 strains showing intense dsRNA bands on polyacrylamide gel electrophoresis (PAGE) analysis were selected (seven strains, KKL-17, PCB-79, PCB-84, PCB-85, PCB-103, SKT-107, and SWL-12, in 2013 (2013 strains), and five strains, NP-130, PCB-656, SKT-457, SSKT-269, and SSL-55, in 2014 (2014 strains)) for full genome-based analysis (data not shown). Fecal specimens were collected during hospital-based surveillance in the RVA vaccine effectiveness evaluation study in Thailand. Stool samples containing the above-mentioned 14 strains were kept at −30°C until use.

### Viral dsRNA Extraction

Extraction of viral dsRNAs was performed as described previously [14, 16, 33]. Briefly, viral dsRNAs were extracted from stool suspensions using a QIAamp Viral RNA Mini Kit (Qiagen). The extracted viral dsRNAs were subjected to Illumina MiSeq sequencing for full genomic analysis as described below.

### cDNA Library Preparation and Illumina MiSeq Sequencing

Building of a cDNA library and Illumina MiSeq sequencing were performed as described previously [14, 16, 30, 33, 34]. Briefly, a 200 bp fragment library ligated with bar-coded adapters was constructed for the 14 strains using an NEBNext Ultra RNA Library Prep Kit for Illumina v1.2 (New England Biolabs), and NEBNext Multiplex Oligos for Illumina (New England Biolabs) according to the manufacturer’s instructions. The cDNA library was purified using Agencourt AMPure XP magnetic beads (Beckman Coulter). After assessing the quality and quantity of the purified cDNA library, nucleotide sequencing was performed on an Illumina MiSeq sequencer (Illumina) using a MiSeq Reagent Kit v2 (Illumina) to generate 151 paired-end reads. Data analysis was performed using CLC Genomics Workbench v8.0.1 (CLC Bio). Contigs were assembled from the obtained sequence reads (trimmed) by *de novo* assembly. Using the assembled contigs as query sequences, the Basic Local Alignment Search Tool (BLAST) non-redundant nucleotide database was searched to obtain the full-length nucleotide sequence of each gene segment of the 14 strains. The nucleotide sequences were translated into amino acid sequences using GENETYX v11 (GENETYX).

### Determination of RVA Genotypes

The genotype of each of the 11 gene segments of the 14 strains was determined using the RotaC v2.0 automated genotyping tool (http://rotac.regatools.be/) [35] according to the guidelines proposed by the Rotavirus Classification Working Group (RCWG).

### Phylogenetic Analysis

Sequence comparisons were performed as described previously [14, 16, 33]. Briefly, multiple alignment of each viral segment was carried out using CLUSTAL W. Phylogenetic trees were constructed using the maximum likelihood method and the Tamura-Nei substitution model using MEGA6.06 [36]. The reliability of the branching order was estimated from 1000 bootstrap replicates. The results of phylogenetic analysis were validated using several other genetic distance models, such as the Hasegawa-Kishino-Yano, Jukes-Cantor, Kimura 2-parameter, and Tamura 3-parameter ones, ruling out any biases among different models concerning the study strains (data not shown).

### Nucleotide Sequence Accession Numbers

The nucleotide sequence data presented in this manuscript have been deposited in the DDBJ and EMBL/GenBank data libraries. The accession numbers for the nucleotide sequences of the VP1-4, VP6, VP7, and NSP1-5 genes of strains KKL-17, PCB-79, PCB-84, PCB-85, PCB-103, SKT-107, SWL-12, NP-130, PCB-656, SKT-457, SSKT-269, SSL-55, LS-202, and LS-L7 are LC169841- LC169851, LC169852- LC169862, LC169863- LC169873, LC169874- LC169884, LC169885- LC169895, LC169896- LC169906, LC169907- LC169917, LC169918- LC169928, LC169929- LC169939, LC169940- LC169950, LC169951- LC169961, LC169962- LC169972, LC169973- LC169983, and LC169984- LC169994, respectively (S1 Table).

## Results

### Nucleotide Sequencing and Full Genome-Based Genotyping

The virion dsRNAs of 12 Thai human G8P[8] strains, KKL-17, PCB-79, PCB-84, PCB-85, PCB-103, SKT-107, SWL-12, NP-130, PCB-656, SKT-457, SSKT-269, and SSL-55, were extracted from stool specimens. To gain an insight into the genetic variability among the 12 strains, the nucleotide sequences of the full genomes of these RVAs were determined using the NGS Illumina MiSeq platform. Furthermore, the full-genomic sequences of two locally circulating human G2P[4] strains, LS-202 and LS-L7, were determined as well, as references. The full genomes of these 14 Thai human strains were amplified using a sequence-independent primer set and then sequenced successfully. Illumina MiSeq sequencing yielded 5.1 x 10^5^ reads (~143 bp average length), 12.9 x 10^5^ reads (~120 bp average length), 12.3 x 10^5^ reads (~138 bp average length), 6.6 x 10^5^ reads (~144 bp average length), 6.8 x 10^5^ reads (~136 bp average length), 7.7 x 10^5^ reads (~140 bp average length), 5.8 x 10^5^ reads (~147 bp average length), 13.0 x 10^5^ reads (~120 bp average length), 12.7 x 10^5^ reads (~121 bp average length), 5.6 x 10^5^ reads (~148 bp average length), 10.7 x 10^5^ reads (~138 bp average length), 8.4 x 10^5^ reads (~145 bp average length), 6.2 x 10^5^ reads (~147 bp average length), and 6.5 x 10^5^ reads (~144 bp average length) for strains KKL-17, PCB-79, PCB-84, PCB-85, PCB-103, SKT-107, SWL-12, NP-130, PCB-656, SKT-457, SSKT-269, SSL-55, LS-202, and LS-L7, respectively. Complete or nearly complete nucleotide sequences of all the 11 genes of the 14 strains could be determined. The lengths of the nucleotide and deduced amino acid sequences of the 11 segments of the 14 strains, with related sequence read data, are summarized in S2 Table.

**Fig 1:**
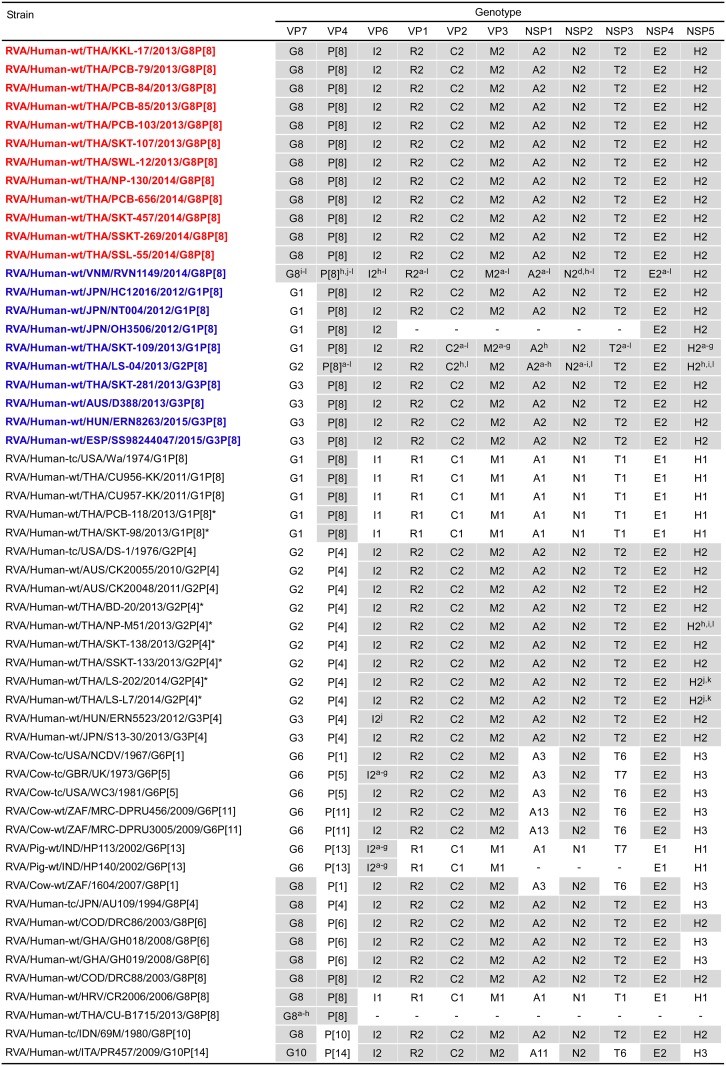
Genotype natures of the 11 gene segments of 12 Thai DS-1-like intergenogroup reassortant strains having G8P[8] genotypes, KKL-17, PCB-79, PCB-84, PCB-85, PCB-103, SKT-107, SWL-12, NP-130, PCB-656, SKT-457, SSKT-269, and SSL-55, compared with those of selected human and animal strains. The 12 study strains are shown in red, while other DS-1-like intergenogroup reassortant strains are shown in blue. Asterisks indicate co-circulating strains, PCB-180, SKT-98, BD-20, NP-M51, SKT-138, SSKT-133, LS-202, and LS-L7, reported in this and the preceding studies [19]. Gray shading indicates the gene segments with genotypes identical to those of the 12 strains. “−” indicates that no sequence data were available in the DDBJ and EMBL/GenBank data libraries. ^a^The gene segments that are most similar to those of strain KKL-17. ^b^The gene segments that are most similar to those of strain PCB-79. ^c^The gene segments that are most similar to those of strain PCB-84. ^d^The gene segments that are most similar to those of strain PCB-85. ^e^The gene segments that are most similar to those of strain PCB-103. ^f^The gene segments that are most similar to those of strain SKT-107. ^g^The gene segments that are most similar to those of strain SWL-12. ^h^The gene segments that are most similar to those of strain NP-130. ^i^The gene segments that are most similar to those of strain PCB-656. ^j^The gene segments that are most similar to those of strain SKT-457. ^k^The gene segments that are most similar to those of strain SSKT-269. ^l^The gene segments that are most similar to those of strain SSL-55.

The 11 genes of the 12 G8P[8] strains were all assigned as G8-P[8]-I2-R2-C2-M2-A2-N2-T2-E2-H2 (Fig 1). The 12 strains were confirmed to have G8P[8] genotypes and a DS-1-like genetic backbone, as indicated on PCR-based G/P genotyping and RNA electropherotyping, respectively [29]. Thus, strains KKL-17, PCB-79, PCB-84, PCB-85, PCB-103, SKT-107, SWL-12, NP-130, PCB-656, SKT-457, SSKT-269, and SSL-55 were named RVA/Human-wt/THA/KKL-17/2013/G8P[8], RVA/Human-wt/THA/PCB-79/2013/G8P[8], RVA/Human-wt/THA/PCB-84/2013/G8P[8], RVA/Human-wt/THA/PCB-85/2013/G8P[8], RVA/Human-wt/THA/PCB-103/2013/G8P[8], RVA/Human-wt/THA/SKT-107/2013/G8P[8], RVA/Human-wt/THA/SWL-12/2013/G8P[8], RVA/Human-wt/THA/NP-130/2014/G8P[8], RVA/Human-wt/THA/PCB-656/2014/G8P[8], RVA/Human-wt/THA/SKT-457/2014/G8P[8], RVA/Human-wt/THA/SSKT-269/2014/G8P[8], and RVA/Human-wt/THA/SSL-55/2014/G8P[8], respectively, according to the guidelines for the uniformity of RVAs proposed by the RCWG. Comparison of the complete genotype constellations of the 12 strains with those of other G8 and non-G8 strains is shown in Fig 1. With the exception of the G genotype, the 12 strains had a unique genotype constellation (P[8]-I2-R2-C2-M2-A2-N2-T2-E2-H2), which is commonly found in DS-1-like intergenogroup reassortant strains [19, 21–27]. Furthermore, the 12 strains exhibited very high nucleotide sequence identities (99.4–100%) to one another for nine of the 11 genes (VP7, VP4, VP1-3, and NSP1-4), the exceptions being the VP6 and NSP5 genes (91.8–100 and 95.8–100%, respectively) (S3 Table). In contrast, the 11 segments of locally circulating strains LS-202 and LS-L7 were assigned as G2-P[4]-I2-R2-C2-M2-A2-N2-T2-E2-H2 (Fig 1), and thus they were named RVA/Human-wt/THA/LS-202/2014/G2P[4] and RVA/Human-wt/THA/LS-L7/2014/G2P[4], respectively. Both the locally circulating strains were shown to possess only genogroup 2 genes. Thus, the complete genotype constellation of co-circulating strains LS-202 and LS-L7 is identical to that of DS-1-like G2P[4] HuRVAs.

### Phylogenetic Analyses

We next constructed phylogenetic trees using the full-length gene sequence for each of the 11 genes because phylogenetic analysis of RVA nucleotide sequences makes it possible to obtain direct evidence of their relatedness to those of other RVA strains, even within the same genotype [17, 19, 26] (Figs 2–14).

**Fig 2:**
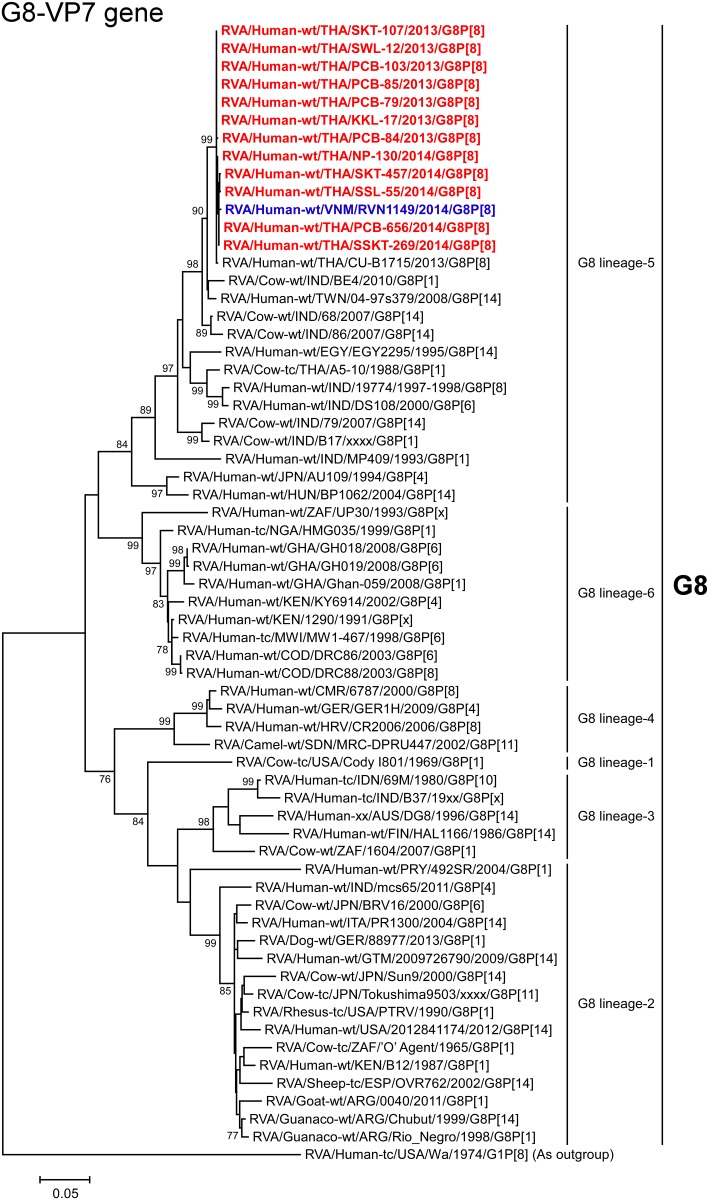
Phylogenetic tree constructed from the nucleotide sequences of the G8-VP7 genes of the study strains and representative RVA strains. In the tree, the positions of the 12 Thai DS-1-like G8P[8] strains are shown in red, while those of other DS-1-like intergenogroup reassortant strains are shown in blue. Asterisks indicate co-circulating strains, PCB-180, SKT-98, BD-20, NP-M51, SKT-138, SSKT-133, LS-202, and LS-07. Bootstrap values of <75% are not shown. Scale bar: 0.05 substitutions per nucleotide.

**Fig 3:**
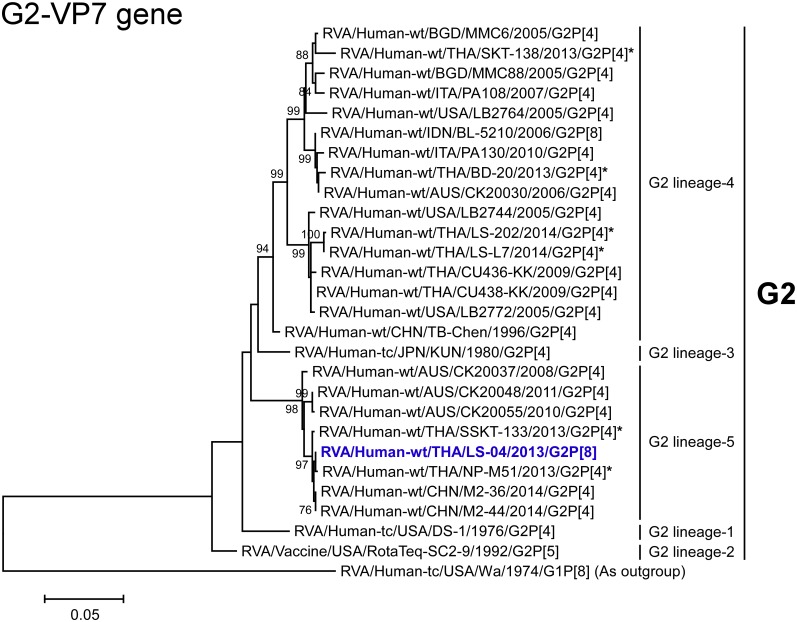
Phylogenetic tree constructed from the nucleotide sequences of the G2-VP7 genes of the study strains and representative RVA strains. See legend to Fig 2. Scale bar: 0.05 substitutions per nucleotide.

**Fig 4:**
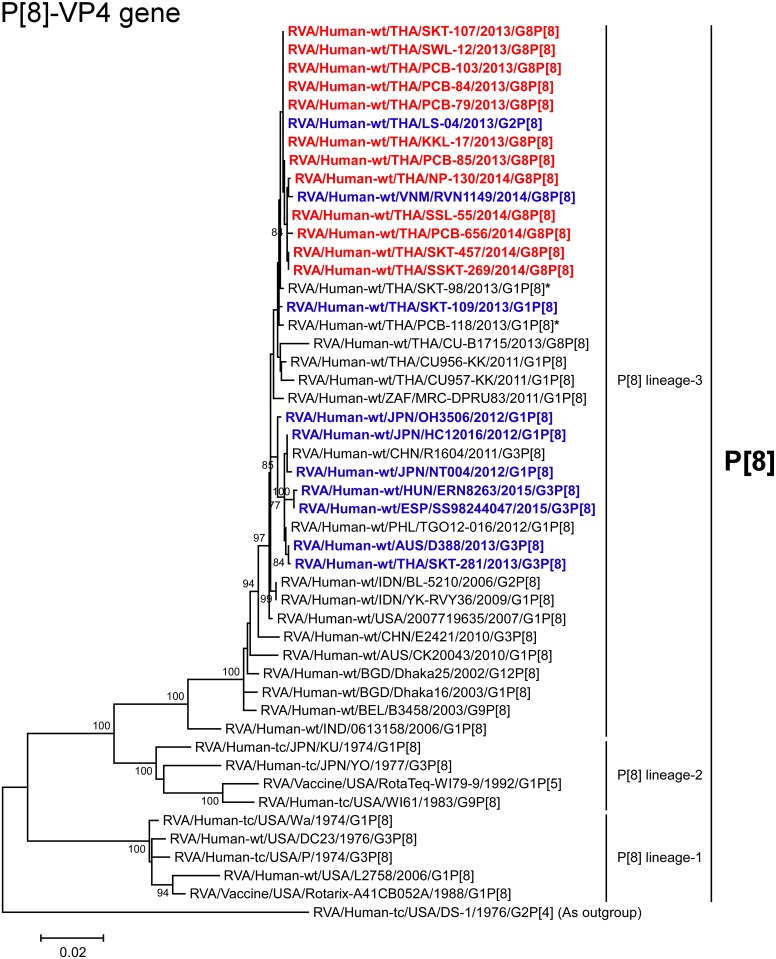
Phylogenetic tree constructed from the nucleotide sequences of the P[8]-VP4 genes of the study strains and representative RVA strains. See legend to Fig 2. Scale bar: 0.02 substitutions per nucleotide.

**Fig 5:**
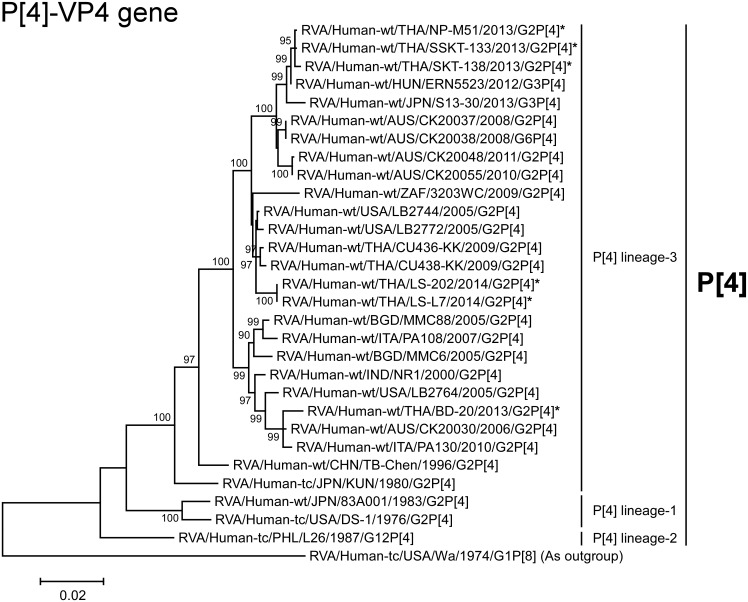
Phylogenetic tree constructed from the nucleotide sequences of the P[4]-VP4 genes of the study strains and representative RVA strains. See legend to Fig 2. Scale bar: 0.02 substitutions per nucleotide.

**Fig 6:**
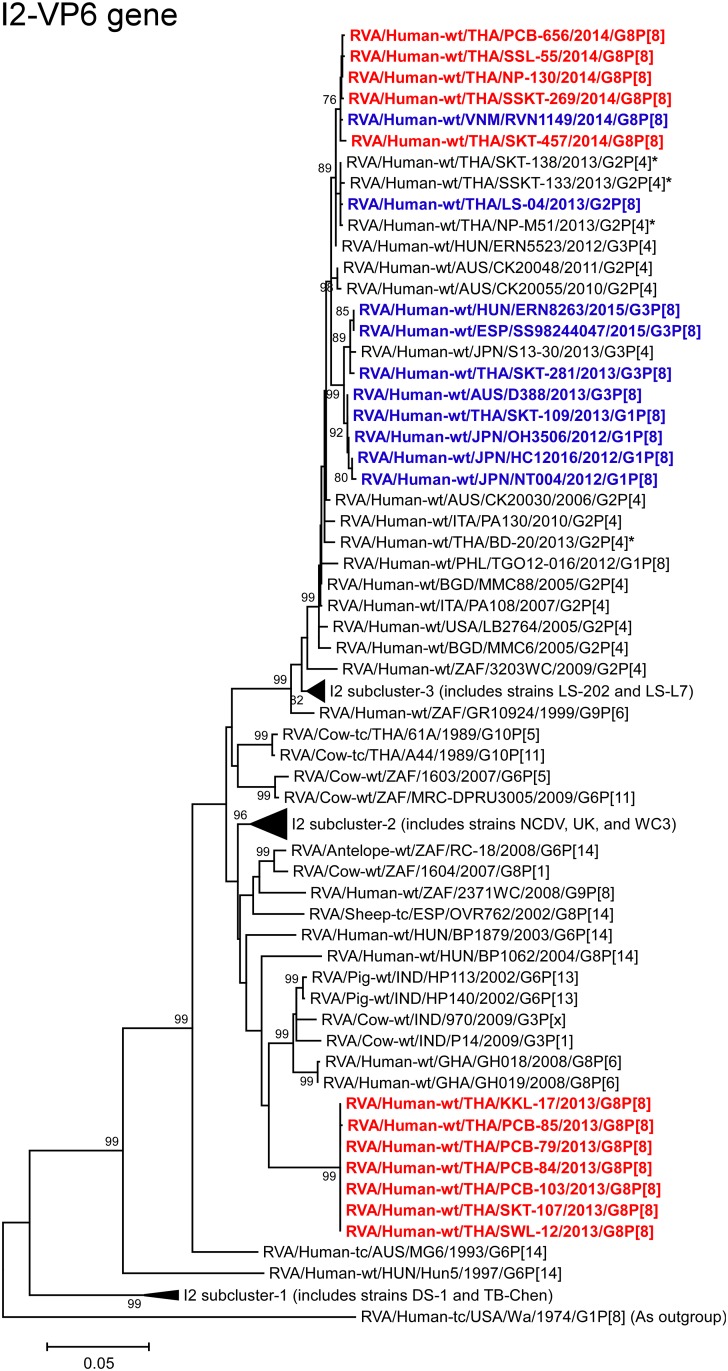
Phylogenetic tree constructed from the nucleotide sequences of the I2-VP6 genes of the study strains and representative RVA strains. See legend to Fig 2. Scale bar: 0.05 substitutions per nucleotide.

**Fig 7:**
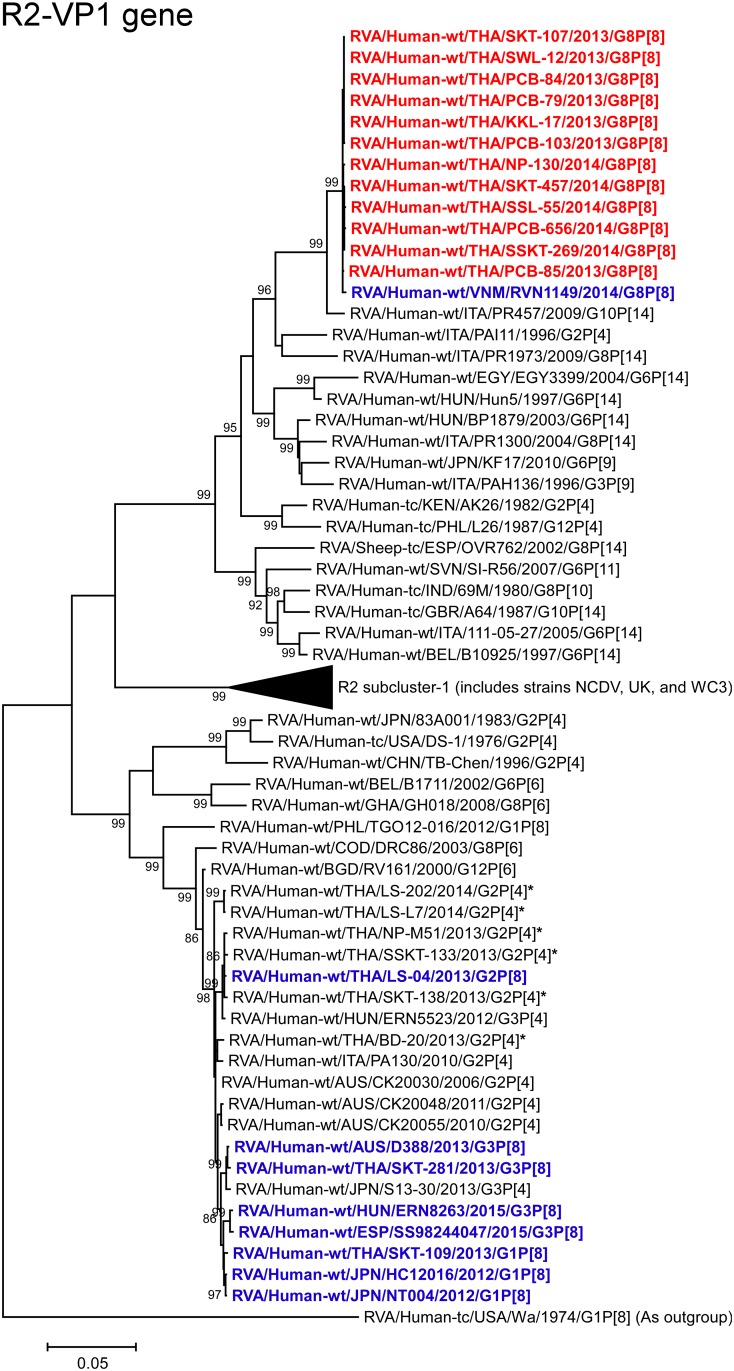
Phylogenetic tree constructed from the nucleotide sequences of the R2-VP1 genes of the study strains and representative RVA strains. See legend to Fig 2. Scale bar: 0.05 substitutions per nucleotide.

**Fig 8:**
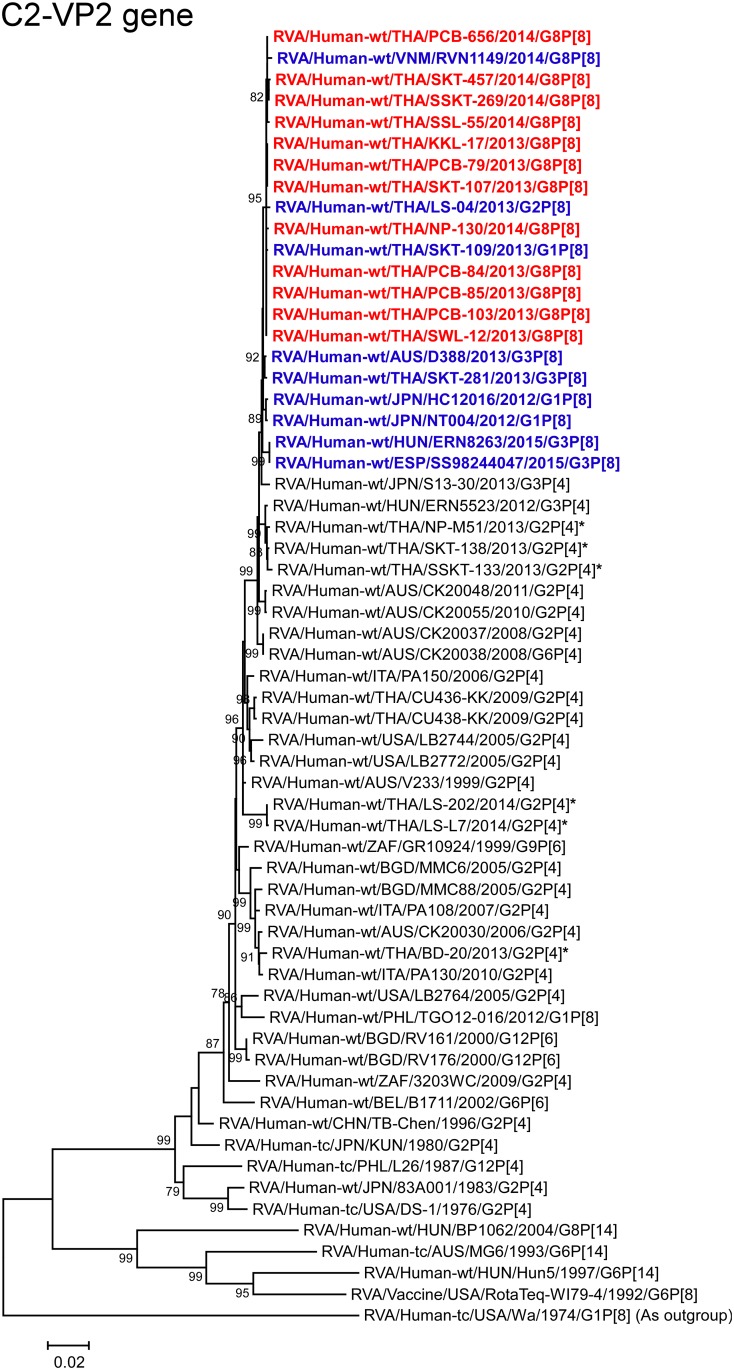
Phylogenetic tree constructed from the nucleotide sequences of the C2-VP2 genes of the study strains and representative RVA strains. See legend to Fig 2. Scale bar: 0.02 substitutions per nucleotide.

**Fig 9:**
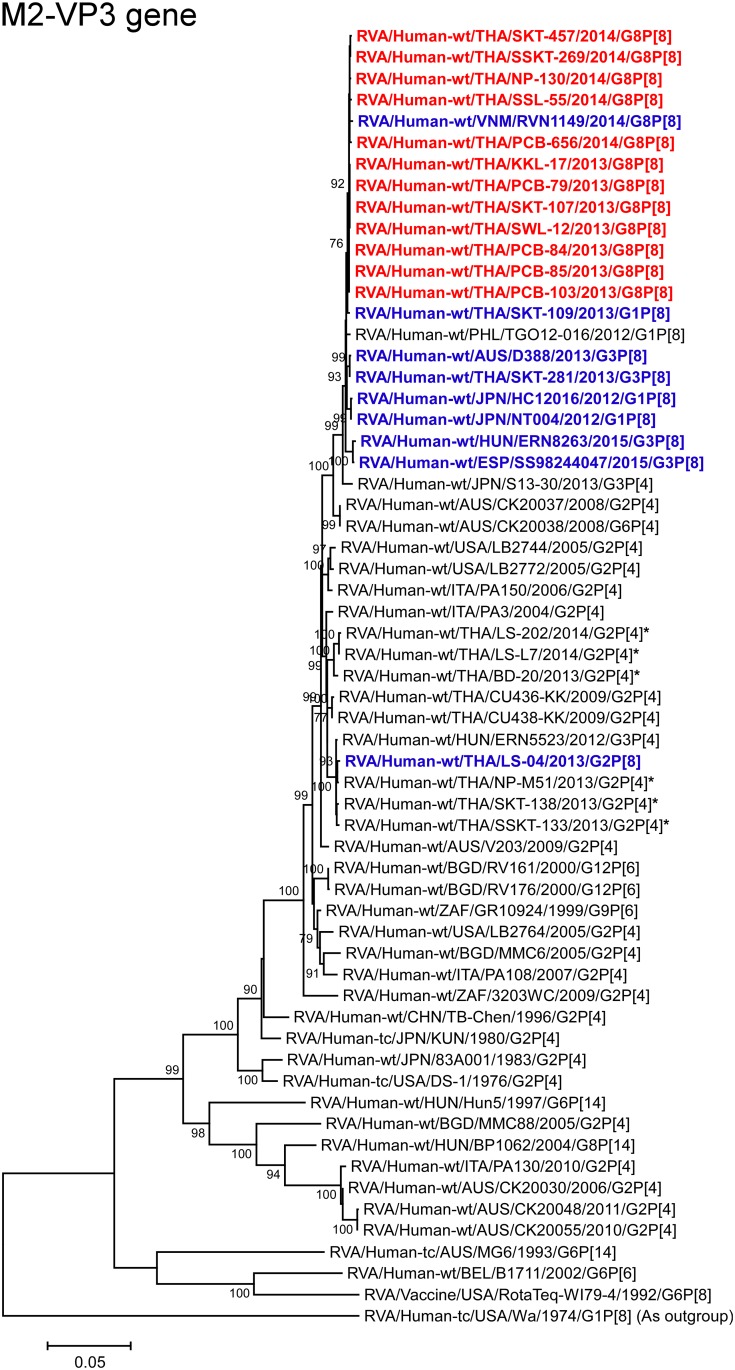
Phylogenetic tree constructed from the nucleotide sequences of the M2-VP3 genes of the study strains and representative RVA strains. See legend to Fig 2. Scale bar: 0.05 substitutions per nucleotide.

**Fig 10:**
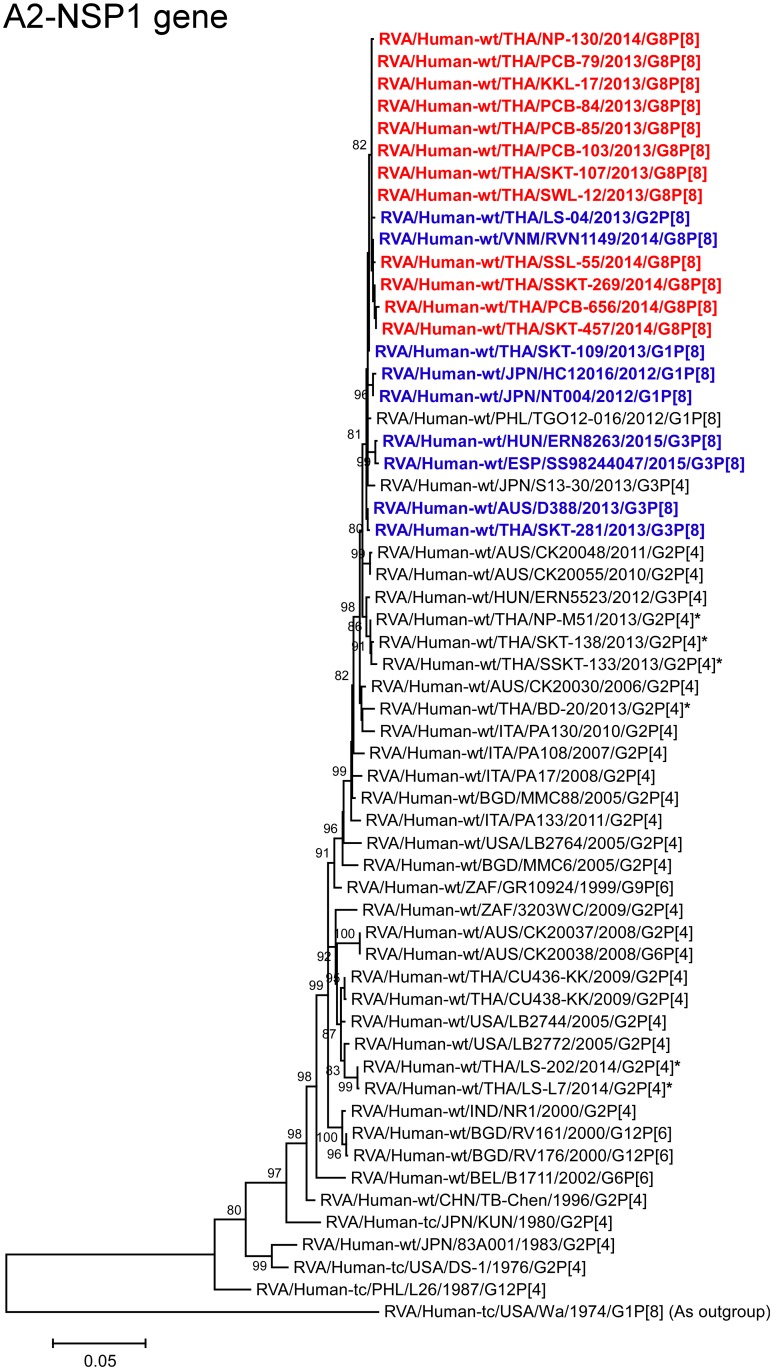
Phylogenetic tree constructed from the nucleotide sequences of the A2-NSP1 genes of the study strains and representative RVA strains. See legend to Fig 2. Scale bar: 0.05 substitutions per nucleotide.

**Fig 11:**
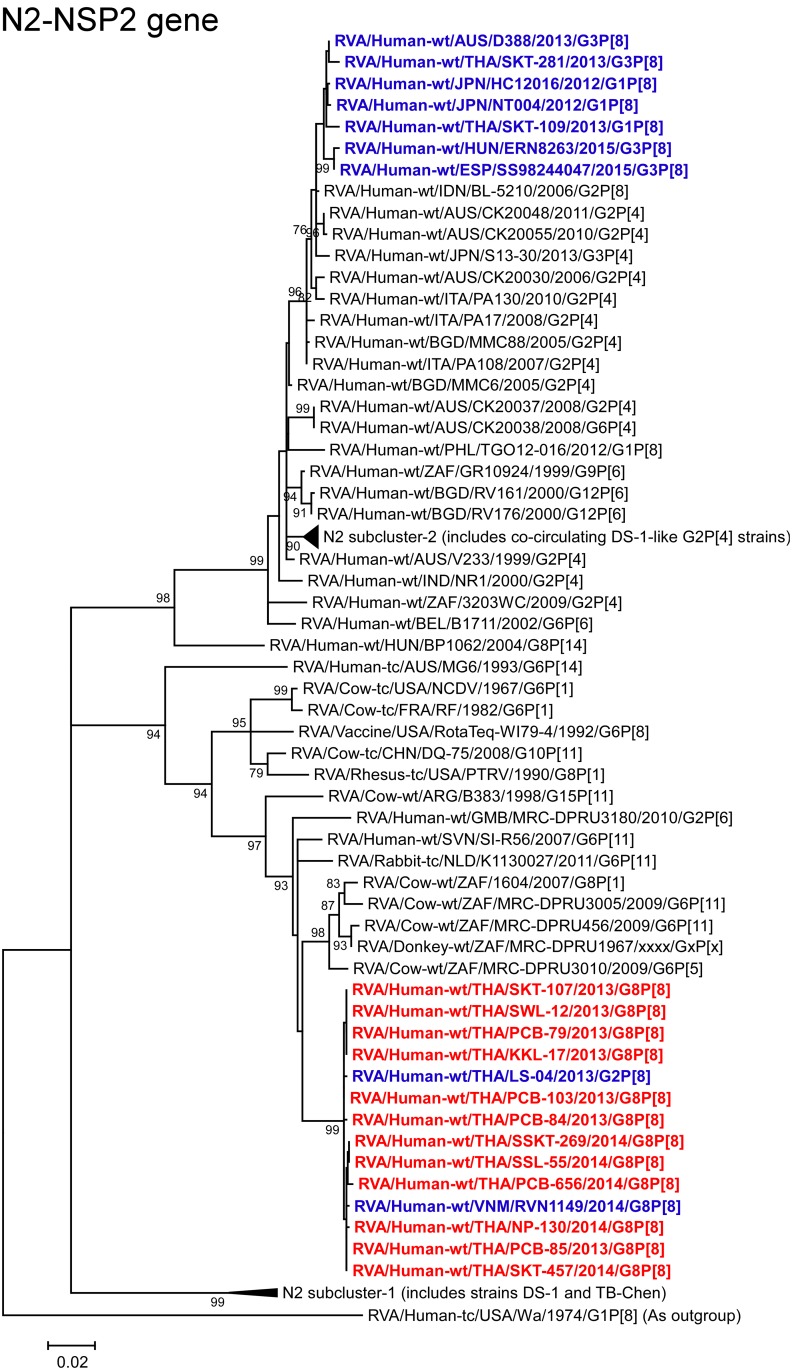
Phylogenetic tree constructed from the nucleotide sequences of the N2-NSP2 genes of the study strains and representative RVA strains. See legend to Fig 2. Scale bar: 0.02 substitutions per nucleotide.

**Fig 12:**
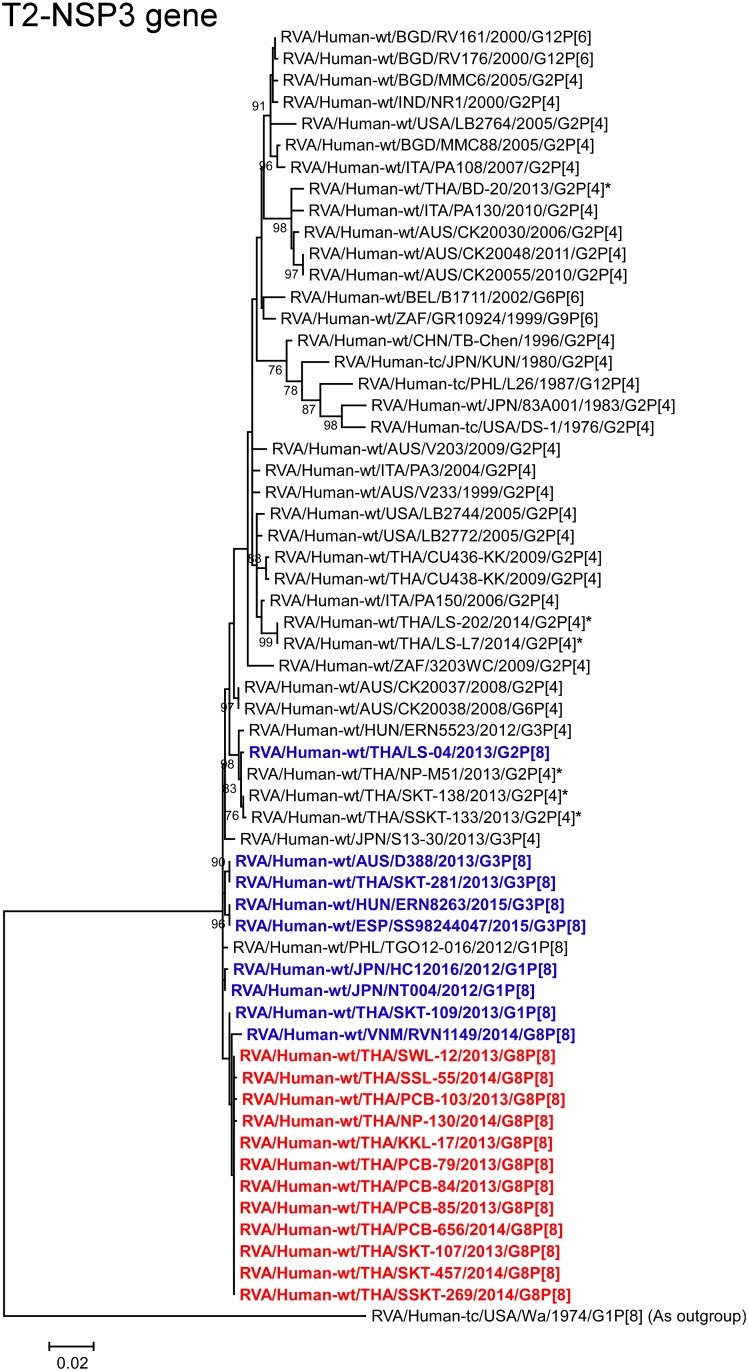
Phylogenetic tree constructed from the nucleotide sequences of the T2-NSP3 genes of the study strains and representative RVA strains. See legend to Fig 2. Scale bar: 0.02 substitutions per nucleotide.

**Fig 13:**
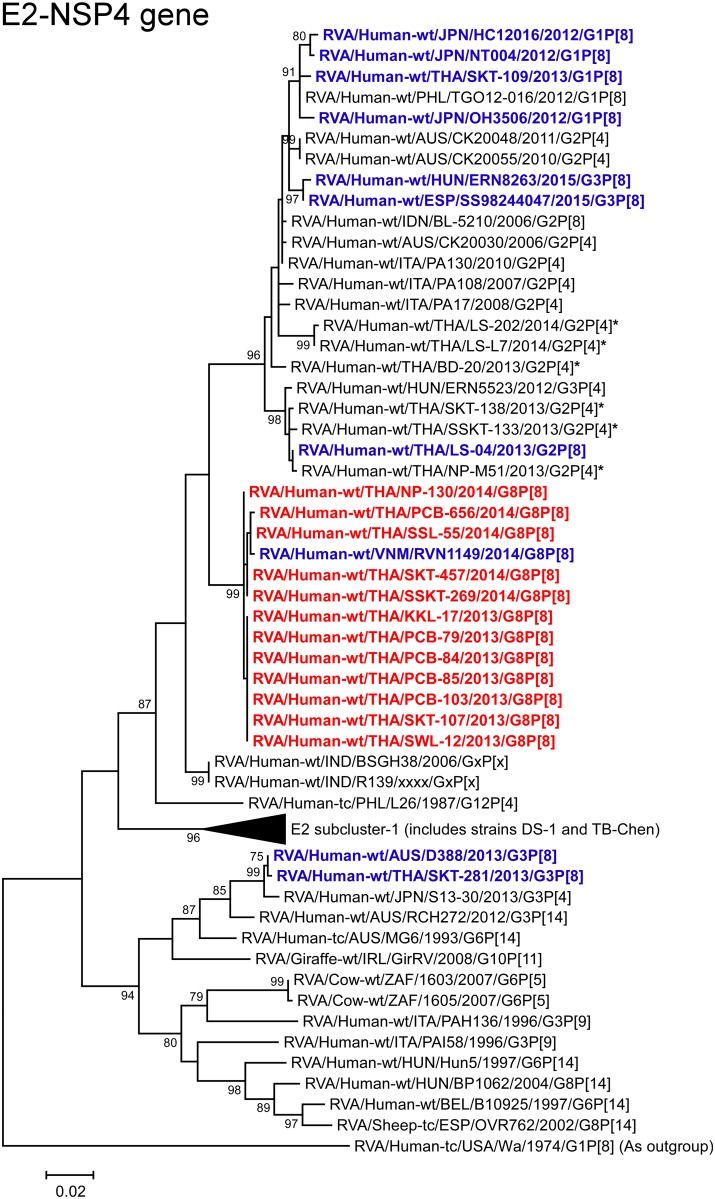
Phylogenetic tree constructed from the nucleotide sequences of the E2-NSP4 genes of the study strains and representative RVA strains. See legend to Fig 2. Scale bar: 0.02 substitutions per nucleotide.

**Fig 14:**
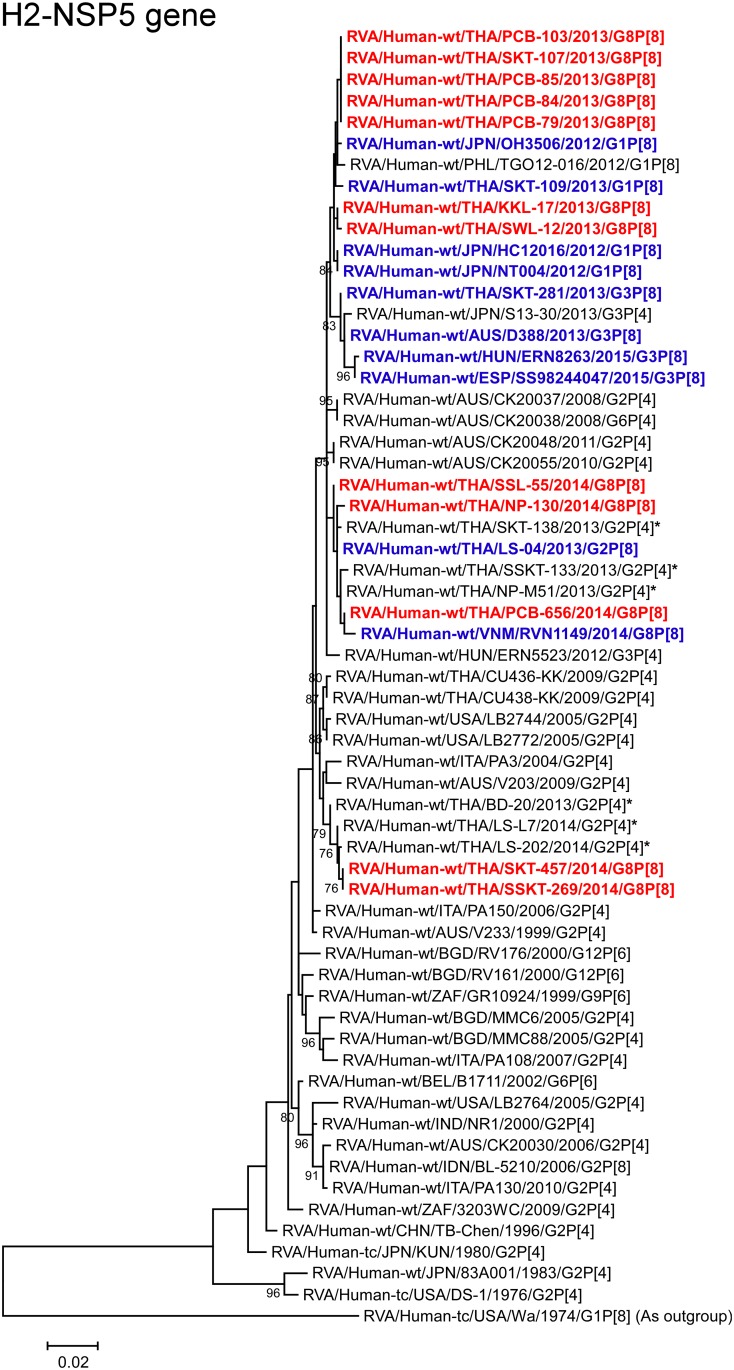
Phylogenetic tree constructed from the nucleotide sequences of the H2-NSP5 genes of the study strains and representative RVA strains. See legend to Fig 2. Scale bar: 0.02 substitutions per nucleotide.

The VP7 genes of all 12 strains exhibited the highest nucleotide sequence identities (99.8–100%) with that of Thai bovine-like human strain CU-B1715 (G8P[8]) [37] or Vietnamese DS-1-like G8P[8] strain RVN1149. On phylogenetic analysis, the 12 strains were shown to form a cluster with the above-mentioned bovine-like human G8P[8] strains from Southeast Asia in a common branch with several bovine and bovine-like human strains from Asia in G8 lineage-5, in which the majority of Asian bovine and bovine-like human strains cluster [38] (Fig 2).

The VP4 genes of all 12 strains exhibited the maximum nucleotide sequence identities (99.7–100%) with the cognate genes of Thai DS-1-like G2P[8] strain LS-04 and/or Vietnamese DS-1-like G8P[8] strain RVN1149. On phylogenetic analysis, the 12 strains were shown to form a cluster with these Southeast Asian DS-1-like intergenogroup reassortant strains and several locally circulating strains having a DS-1-like or Wa-like genetic backbone in human-like P[8] lineage-3 (Fig 4).

The VP6 genes of the 2013 strains (KKL-17, PCB-79, PCB-84, PCB-85, PCB-103, SKT-107, and SWL-12) showed the maximum nucleotide sequence identities (94.8–94.9%) with the VP6 genes of Indian bovine-like porcine strains HP113 (G6P[13]) and HP140 (G6P[13]) [39, 40]. Phylogenetically, the 2013 strains were found to form a cluster near the cluster comprising strains HP113 and HP140, and several bovine and bovine-like human strains within the bovine-like I2 subcluster (Fig 6). On the other hand, the VP6 genes of the 2014 strains (NP-130, PCB-656, SKT-457, SSKT-269, and SSL-55) exhibited the highest nucleotide sequence identities (99.6–99.8%) with Vietnamese DS-1-like G8P[8] strain RVN1149. Phylogenetically, the 2014 strains formed a cluster with strain RVN1149 near the cluster with co-circulating DS-1-like G2P[4] strains NP-M51, SKT-138, and SSKT-133 [19], and Thai DS-1-like G2P[8] strain LS-04 within the human-like I2 subcluster (Fig 6).

The VP1, VP3, and NSP4 genes of all 12 strains exhibited the highest nucleotide sequence identities (99.7–99.8, 99.8, and 99.5–99.8%, respectively) with those of Vietnamese DS-1-like G8P[8] strain RVN1149. Phylogenetically, the 12 strains were found to form a cluster with strain RVN1149 within the human-like R2, M2, and E2 subclusters (Figs 7, 9 and 13).

The VP2 genes of all 12 strains exhibited the maximum nucleotide sequence identities (99.8–99.9%) with those of Thai DS-1-like G1P[8] strain SKT-109 and/or Thai DS-1-like G2P[8] strain LS-04, and comparable identities (99.6–99.8%) with Vietnamese DS-1-like G8P[8] strain RVN1149. On phylogenetic analysis, the 12 strains were shown to form a cluster with these Southeast Asian DS-1-like intergenogroup reassortant strains near the cluster consisting of the other DS-1-like intergenogroup reassortant strains within the human-like C2 subcluster (Fig 8).

The NSP1 genes of all 12 strains showed the maximum nucleotide sequence identities (99.8–99.9%) with those of Thai DS-1-like G2P[8] strain LS-04 and/or Vietnamese DS-1-like G8P[8] strain RVN1149. Phylogenetically, the 12 strains were shown to form a cluster with these Southeast Asian DS-1-like intergenogroup reassortant strains near the cluster with the other DS-1-like intergenogroup reassortant strains within the human-like A2 subcluster (Fig 10).

The NSP2 genes of all 12 strains showed the maximum nucleotide sequence identities (99.7–99.9%) with the cognate genes of Thai DS-1-like G2P[8] strain LS-04 and/or Vietnamese DS-1-like G8P[8] strain RVN1149. Phylogenetically, the 12 strains were found to form a distinct cluster with these Southeast Asian DS-1-like intergenogroup reassortant strains in a common branch with several bovine and bovine-like donkey strains from South Africa within the bovine-like N2 subcluster (Fig 11).

The NSP3 genes of all 12 strains exhibited the maximum nucleotide sequence identities (99.7–99.9%) with that of Thai DS-1-like G1P[8] strain SKT-109. On phylogenetic analysis, the 12 strains were shown to form a cluster with strain SKT-109 and Vietnamese DS-1-like G8P[8] strain RVN1149 within the human-like T2 subcluster (Fig 12).

The NSP5 genes of the 2013 strains (KKL-17, PCB-79, PCB-84, PCB-85, PCB-103, SKT-107, and SWL-12) showed the maximum nucleotide sequence identities (99.6–99.7%) with those of Thai DS-1-like G1P[8] strain SKT-109, and Japanese DS-1-like G1P[8] strains HC12016, NT004, and OH3506. Phylogenetically, the 2013 strains were shown to form a cluster with these DS-1-like G1P[8] strains (Fig 14). On the other hand, the NSP5 genes of three of the 2014 strains (NP-130, PCB-656, and SSL-55) showed the highest nucleotide sequence identities (99.7–99.8%) with locally circulating DS-1-like G2P[4] strain NP-M51 and Thai DS-1-like G2P[8] strain LS-04. Phylogenetically, strains NP-130, PCB-656, and SSL-55 were shown to form a cluster with these Thai human G2 strains, locally circulating DS-1-like G2P[4] strains (SKT-138 and SSKT-133), and Vietnamese DS-1-like G8P[8] strain RVN1149 (Fig 14). Moreover, the NSP5 genes of the remaining two 2014 strains (SKT-457 and SSKT-269) showed the highest nucleotide sequence identities (97.5%) with locally circulating DS-1-like G2P[4] strains LS-202 and LS-L7. Phylogenetically, strains SKT-457 and SSKT-269 were shown to be closely related with strains LS-202 and LS-L7 (Fig 14).

## Discussion

In the present study, we analyzed the full genomes of 12 DS-1-like intergenogroup reassortant strains having G8P[8] genotypes (seven strains in 2013 and five strains in 2014) identified in stool specimens from hospitalized children with severe diarrhea in Thailand. All 12 strains exhibited a unique genotype constellation comprising a mixture of genogroup 1 and 2 genes: G8-P[8]-I2-R2-C2-M2-A2-N2-T2-E2-H2. With the exception of the G genotype, the unique genotype constellation of the 12 strains (P[8]-I2-R2-C2-M2-A2-N2-T2-E2-H2) is commonly shared with DS-1-like intergenogroup reassortant strains. On phylogenetic analysis, six of the 11 genes of the 2013 strains (VP4, VP2, VP3, NSP1, NSP3, and NSP5) appeared to have originated from DS-1-like intergenogroup reassortant strains, while the remaining four (VP7, VP6, VP1, and NSP2) and one (NSP4) were assumed to be of bovine and human origin, respectively. Therefore, the 2013 strains seemed to have been derived through reassortment involving DS-1-like intergenogroup reassortant, bovine, bovine-like human, and/or human rotaviruses. On the other hand, five of the 11 genes of the 2014 strains (VP4, VP2, VP3, NSP1, and NSP3) were assumed to have originated from DS-1-like intergenogroup reassortant strains, while three (VP7, VP1, and NSP2) and one (NSP4) appeared to be of bovine and human origin, respectively. Of note is that the remaining VP6 and NSP5 genes of the 2014 strains were assumed to have originated from co-circulating DS-1-like G2P[4] HuRVAs. Therefore, the 2014 strains appeared to be multiple reassortment strains involving DS-1-like intergenogroup reassortant, bovine, bovine-like human, human, and/or locally circulating DS-1-like G2P[4] rotaviruses. These results might imply the occurrence of reassortment between the 2013 strains and locally circulating DS-1-like G2P[4] strains to form the 2014 strains having the VP6 and NSP5 genes derived from co-circulating DS-1-like G2P[4] rotaviruses. Overall, the great genomic diversity among the DS-1-like intergenogroup reassortant strains seemed to have been generated through additional reassortment involving animal and human strains. However, the exact origins of the bovine-like VP7, VP6, VP1, and/or NSP2 genes, and human-like NSP4 genes of the 12 strains could not be ascertained due to a lack of a sufficient number of representative bovine-like human, bovine, and human strains as references. In any case, our findings add the increasing evidence supporting animal-to-human interspecies transmission through reassortment [7]. The bovine origin of the 12 strains suggests interspecies transmission due to the close proximity between humans and livestock, especially in developing countries in Asia, where there is intimate contact between humans and livestock such as cattle in daily life [14].

Of note is that all the 11 segments of three of the 2014 strains, NP-130, PCB-656, and SSL-55, were very closely related to those of Vietnamese DS-1-like G8P[8] strains that emerged in 2014–2015, indicating the derivation of these DS-1-like G8P[8] strains from a common ancestor. In contrast, the low genomic correlation between Asian DS-1-like G8P[8] strains and African ones indicates the distinct evolution of DS-1-like G8P[8] strains in Asia and Africa. However, a global rotavirus strain collection is required to determine the exact evolutionary dynamics of the emerging DS-1-like G8P[8] strains in Asia. To our knowledge, this is the first report on full genome-based analysis of DS-1-like G8P[8] strains that have emerged in Thailand. It is essential to continue monitoring of the emergence of any uncommon RVA variants such as DS-1-like intergenogroup reassortant strains in order to correlate the relationship with the current introduction of HuRVA vaccines [19].

The emergence of DS-1-like G8P[8] strains in Thailand may imply the constant circulation of DS-1-like intergenogroup reassortant strains and the occurrence of reassortment involving them in Asia. Because DS-1-like intergenogroup reassortant strains have successfully spread in multiple locations in Asia, Australia, and Europe in a short period [19, 21–27], continued monitoring of DS-1-like intergenogroup reassortant strains is required. Although most investigations on RVA genotype distributions have been focused on only G/P defining genes, PCR-based genotyping for non-G/P defining gene(s) or PAGE analysis will assist the recognition of novel RVA variants such as DS-1-like intergenogroup reassortant strains, as described in the preceding study [19]. Furthermore, full genome-based analysis is a reliable tool to understand the emergence and evolution of DS-1-like intergenogroup reassortant strains [19].

## Supporting Information

S1 Table The DDBJ and EMBL/GenBank accession numbers of 14 Thai RVA strains, KKL-17, PCB-79, PCB-84, PCB-85, PCB-103, SKT-107, SWL-12, NP-130, PCB-656, SKT-457, SSKT-269, SSL-55, LS-202, and LS-L7. (DOCX)

S2 Table Sequence data for the 11 gene segments of 14 Thai RVA strains, KKL-17, PCB-79, PCB-84, PCB-85, PCB-103, SKT-107, SWL-12, NP-130, PCB-656, SKT-457, SSKT-269, SSL-55, LS-202, and LS-L7. (DOCX)

S3 Table Nucleotide sequence identities (%) of the 11 gene segments of 12 Thai DS-1-like intergenogroup reassortant strains having G8P[8] genotypes, KKL-17, PCB-79, PCB-84, PCB-85, PCB-103, SKT-107, SWL-12, NP-130, PCB-656, SKT-457, SSKT-269, and SSL-55, to one another. (DOCX)
