# Supplementary material for: Full Genome Characterization of Novel DS-1-Like G8P[8] Rotavirus Strains that Have Emerged in Thailand: Reassortment of Bovine and Human Rotavirus Gene Segments in Emerging DS-1-Like Intergenogroup Reassortant Strains
Source: PLoS One. 2016 Nov 1;11(11):e0165826. doi: 10.1371/journal.pone.0165826 (PMC5089778; doi:10.1371/journal.pone.0165826)
Supplement: S1 Table — (DOCX) [file pone.0165826.s001.docx]

**S1 Table.** The DDBJ and EMBL/GenBank accession numbers of 14 Thai strains, KKL-17, PCB-79, PCB-84, PCB-85, PCB-103, SKT-107, SWL-12, NP-130, PCB-656, SKT-457, SSKT-269, SSL-55, LS-202, and LS-L7.

| Study strain | Gene | | | | | | | | | | |
| --- | --- | --- | --- | --- | --- | --- | --- | --- | --- | --- | --- |
|  | VP1 | VP2 | VP3 | VP4 | VP6 | VP7 | NSP1 | NSP2 | NSP3 | NSP4 | NSP5 |
| RVA/Human-wt/THA/KKL-17/2013/G8P[8] | LC169841 | LC169842 | LC169843 | LC169844 | LC169845 | LC169846 | LC169847 | LC169848 | LC169849 | LC169850 | LC169851 |
| RVA/Human-wt/THA/PCB-79/2013/G8P[8] | LC169852 | LC169853 | LC169854 | LC169855 | LC169856 | LC169857 | LC169858 | LC169859 | LC169860 | LC169861 | LC169862 |
| RVA/Human-wt/THA/PCB-84/2013/G8P[8] | LC169863 | LC169864 | LC169865 | LC169866 | LC169867 | LC169868 | LC169869 | LC169870 | LC169871 | LC169872 | LC169873 |
| RVA/Human-wt/THA/PCB-85/2013/G8P[8] | LC169874 | LC169875 | LC169876 | LC169877 | LC169878 | LC169879 | LC169880 | LC169881 | LC169882 | LC169883 | LC169884 |
| RVA/Human-wt/THA/PCB-103/2013/G8P[8] | LC169885 | LC169886 | LC169887 | LC169888 | LC169889 | LC169890 | LC169891 | LC169892 | LC169893 | LC169894 | LC169895 |
| RVA/Human-wt/THA/SKT-107/2013/G8P[8] | LC169896 | LC169897 | LC169898 | LC169899 | LC169900 | LC169901 | LC169902 | LC169903 | LC169904 | LC169905 | LC169906 |
| RVA/Human-wt/THA/SWL-12/2013/G8P[8] | LC169907 | LC169908 | LC169909 | LC169910 | LC169911 | LC169912 | LC169913 | LC169914 | LC169915 | LC169916 | LC169917 |
| RVA/Human-wt/THA/NP-130/2014/G8P[8] | LC169918 | LC169919 | LC169920 | LC169921 | LC169922 | LC169923 | LC169924 | LC169925 | LC169926 | LC169927 | LC169928 |
| RVA/Human-wt/THA/PCB-656/2014/G8P[8] | LC169929 | LC169930 | LC169931 | LC169932 | LC169933 | LC169934 | LC169935 | LC169936 | LC169937 | LC169938 | LC169939 |
| RVA/Human-wt/THA/SKT-457/2014/G8P[8] | LC169940 | LC169941 | LC169942 | LC169943 | LC169944 | LC169945 | LC169946 | LC169947 | LC169948 | LC169949 | LC169950 |
| RVA/Human-wt/THA/SSKT-269/2014/G8P[8] | LC169951 | LC169952 | LC169953 | LC169954 | LC169955 | LC169956 | LC169957 | LC169958 | LC169959 | LC169960 | LC169961 |
| RVA/Human-wt/THA/SSL-55/2014/G8P[8] | LC169962 | LC169963 | LC169964 | LC169965 | LC169966 | LC169967 | LC169968 | LC169969 | LC169970 | LC169971 | LC169972 |
| RVA/Human-wt/THA/LS-202/2014/G2P[4] | LC169973 | LC169974 | LC169975 | LC169976 | LC169977 | LC169978 | LC169979 | LC169980 | LC169981 | LC169982 | LC169983 |
| RVA/Human-wt/THA/LS-L7/2014/G2P[4] | LC169984 | LC169985 | LC169986 | LC169987 | LC169988 | LC169989 | LC169990 | LC169991 | LC169992 | LC169993 | LC169994 |
