# Supplementary material for: Full Genome Characterization of Novel DS-1-Like G8P[8] Rotavirus Strains that Have Emerged in Thailand: Reassortment of Bovine and Human Rotavirus Gene Segments in Emerging DS-1-Like Intergenogroup Reassortant Strains
Source: PLoS One. 2016 Nov 1;11(11):e0165826. doi: 10.1371/journal.pone.0165826 (PMC5089778; doi:10.1371/journal.pone.0165826)
Supplement: S2 Table — (DOCX) [file pone.0165826.s002.docx]

**S2 Table.** Sequence data for the 11 gene segments of 14 Thai RVA strains, KKL-17, PCB-79, PCB-84, PCB-85, PCB-103, SKT-107, SWL-12, NP-130, PCB-656, SKT-457, SSKT-269, SSL-55, LS-202, and LS-L7.

| Study strain | Total reads^a^ |  | Gene | | | | | | | | | | |
| --- | --- | --- | --- | --- | --- | --- | --- | --- | --- | --- | --- | --- | --- |
|  |  |  | VP7 | VP4 | VP6 | VP1 | VP2 | VP3 | NSP1 | NSP2 | NSP3 | NSP4 | NSP5 |
| RVA/Human-wt/THA/KKL-17/2013/G8P[8] | 511,860 | Nucleotides; bp  (% coverage of the full-length) | 1062  (100%) | 2359  (100%) | 1355  (99.9%) | 3302  (100%) | 2677  (99.7%) | 2591  (100%) | 1566  (100%) | 1047  (98.9%) | 1066  (100%) | 750  (99.9%) | 803  (98.4%) |
|  |  | Deduced amino acids; aa  (% coverage of the full-length) | 326  (100%) | 775  (100%) | 397  (100%) | 1088  (100%) | 879  (100%) | 835  (100%) | 486  (100%) | 317  (100%) | 313  (100%) | 175  (100%) | 200  (100%) |
|  |  | Reads mapped to gene segment  (% on-target reads of the total reads) | 32,406  (6.3%) | 71,686  (14.0%) | 17,678  (3.5%) | 99,540  (19.4%) | 84,073  (16.4%) | 72,016  (14.1%) | 28,601  (5.6%) | 25,074  (4.9%) | 31,812  (6.2%) | 15,726  (3.1%) | 12,646  (2.5%) |
|  |  | Maximum depth of reads | 5,948 | 6,006 | 4,704 | 4,475 | 4,925 | 3,955 | 4,095 | 3,870 | 4,048 | 4,302 | 3,799 |
| RVA/Human-wt/THA/PCB-79/2013/G8P[8] | 1,294,950 | Nucleotide; bp  (% coverage of the full-length) | 1062  (100%) | 2359  (100%) | 1356  (100%) | 3302  (100%) | 2677  (99.7%) | 2591  (100%) | 1566  (100%) | 1059  (100%) | 1066  (100%) | 740  (98.5%) | 816  (100%) |
|  |  | Deduced amino acids; aa  (% coverage of the full-length) | 326  (100%) | 775  (100%) | 397  (100%) | 1088  (100%) | 879  (100%) | 835  (100%) | 486  (100%) | 317  (100%) | 313  (100%) | 175  (100%) | 200  (100%) |
|  |  | Reads mapped to gene segment  (% on-target reads of the total reads) | 85,754  (6.6%) | 109,800  (8.5%) | 26,861  (2.1%) | 93,461  (7.2%) | 145,568  (11.2%) | 95,354  (7.4%) | 36,646  (2.8%) | 44,003  (3.4%) | 57,315  (4.4%) | 17,811  (1.4%) | 12,257  (0.9%) |
|  |  | Maximum depth of reads | 11,458 | 6,629 | 6,273 | 6,517 | 11,607 | 5,084 | 7,307 | 7,945 | 6,413 | 7,513 | 3,732 |
| RVA/Human-wt/THA/PCB-84/2013/G8P[8] | 1,231,060 | Nucleotide; bp  (% coverage of the full-length) | 1062  (100%) | 2359  (100%) | 1356  (100%) | 3302  (100%) | 2684  (100%) | 2591  (100%) | 1566  (100%) | 1050  (99.2%) | 1066  (100%) | 737  (98.1%) | 816  (100%) |
|  |  | Deduced amino acids; aa  (% coverage of the full-length) | 326  (100%) | 775  (100%) | 397  (100%) | 1088  (100%) | 879  (100%) | 835  (100%) | 486  (100%) | 317  (100%) | 313  (100%) | 175  (100%) | 200  (100%) |
|  |  | Reads mapped to gene segment  (% on-target reads of the total reads) | 66,247  (5.4%) | 160,871  (13.1%) | 71,506  (5.8%) | 219,508  (17.8%) | 190,387  (15.5%) | 153,675  (12.5%) | 60,426  (4.9%) | 56,892  (4.6%) | 63,982  (5.2%) | 39,064  (3.2%) | 29,009  (2.4%) |
|  |  | Maximum depth of reads | 10,483 | 12,041 | 9,468 | 9,779 | 11,308 | 8,279 | 8,252 | 7,863 | 7,436 | 11,051 | 7,324 |
| RVA/Human-wt/THA/PCB-85/2013/G8P[8] | 659,350 | Nucleotide; bp  (% coverage of the full-length) | 1062  (100%) | 2359  (100%) | 1350  (99.6%) | 3298  (99.9%) | 2674  (99.6%) | 2591  (100%) | 1566  (100%) | 1049  (99.1%) | 1066  (100%) | 751  (100%) | 805  (98.7%) |
|  |  | Deduced amino acids; aa  (% coverage of the full-length) | 326  (100%) | 775  (100%) | 397  (100%) | 1088  (100%) | 879  (100%) | 835  (100%) | 486  (100%) | 317  (100%) | 313  (100%) | 175  (100%) | 200  (100%) |
|  |  | Reads mapped to gene segment  (% on-target reads of the total reads) | 40,941  (6.2%) | 81,997  (12.4%) | 17,238  (2.6%) | 112,104  (17.0%) | 105,232  (16.0%) | 91,387  (13.9%) | 39,875  (6.0%) | 108,952  (16.5%) | 43,059  (6.5%) | 7,573  (1.1%) | 5,371  (0.8%) |
|  |  | Maximum depth of reads | 6,801 | 5,328 | 5,474 | 6,248 | 6,284 | 5,200 | 4,674 | 6,302 | 5,682 | 2,507 | 1,031 |
| RVA/Human-wt/THA/PCB-103/2013/G8P[8] | 679,896 | Nucleotide; bp  (% coverage of the full-length) | 1062  (100%) | 2359  (100%) | 1355  (99.9%) | 3296  (99.8%) | 2674  (99.6%) | 2591  (100%) | 1566  (100%) | 1047  (98.9%) | 1066  (100%) | 745  (99.2%) | 812  (99.5%) |
|  |  | Deduced amino acids; aa  (% coverage of the full-length) | 326  (100%) | 775  (100%) | 397  (100%) | 1088  (100%) | 879  (100%) | 835  (100%) | 486  (100%) | 317  (100%) | 313  (100%) | 175  (100%) | 200  (100%) |
|  |  | Reads mapped to gene segment  (% on-target reads of the total reads) | 46,913  (6.9%) | 100,210  (14.7%) | 23,031  (3.4%) | 125,290  (18.4%) | 113,532  (16.7%) | 92,746  (13.6%) | 35,624  (5.2%) | 35,793  (5.3%) | 41,064  (6.0%) | 19,541  (2.9%) | 14,076  (2.1%) |
|  |  | Maximum depth of reads | 7,994 | 8,278 | 6,251 | 5,087 | 8,028 | 5,010 | 6,037 | 5,397 | 4,532 | 4,666 | 3,619 |
| RVA/Human-wt/THA/SKT-107/2013/G8P[8] | 768,082 | Nucleotide; bp  (% coverage of the full-length) | 1062  (100%) | 2359  (100%) | 1351  (99.6%) | 3302  (100%) | 2677  (99.7%) | 2591  (100%) | 1566  (100%) | 1047  (98.9%) | 1066  (100%) | 732  (97.5%) | 816  (100%) |
|  |  | Deduced amino acids; aa  (% coverage of the full-length) | 326  (100%) | 775  (100%) | 397  (100%) | 1088  (100%) | 879  (100%) | 835  (100%) | 486  (100%) | 317  (100%) | 313  (100%) | 175  (100%) | 200  (100%) |
|  |  | Reads mapped to gene segment  (% on-target reads of the total reads) | 27,709  (3.6%) | 61,453  (8.0%) | 13,315  (1.7%) | 71,462  (9.3%) | 67,041  (8.7%) | 58,864  (7.7%) | 25,440  (3.3%) | 20,771  (2.7%) | 25,583  (3.3%) | 7,771  (1.0%) | 6,770  (0.9%) |
|  |  | Maximum depth of reads | 4,838 | 4,983 | 3,565 | 3,308 | 3,656 | 3,066 | 3,155 | 2,984 | 3,189 | 1,949 | 1,701 |
| RVA/Human-wt/THA/SWL-12/2013/G8P[8] | 582,642 | Nucleotide; bp  (% coverage of the full-length) | 1062  (100%) | 2359  (100%) | 1356  (100%) | 3302  (100%) | 2684  (100%) | 2591  (100%) | 1566  (100%) | 1049  (99.1%) | 1066  (100%) | 740  (98.5%) | 808  (99.0%) |
|  |  | Deduced amino acids; aa  (% coverage of the full-length) | 326  (100%) | 775  (100%) | 397  (100%) | 1088  (100%) | 879  (100%) | 835  (100%) | 486  (100%) | 317  (100%) | 313  (100%) | 175  (100%) | 200  (100%) |
|  |  | Reads mapped to gene segment  (% on-target reads of the total reads) | 36,197  (6.2%) | 83,175  (14.3%) | 19,400  (3.3%) | 111,262  (19.1%) | 92,540  (15.9%) | 80,095  (13.7%) | 34,942  (6.0%) | 30,197  (5.2%) | 36,130  (6.2%) | 19,089  (3.3%) | 15,830  (2.7%) |
|  |  | Maximum depth of reads | 6,484 | 6,986 | 5,168 | 5,581 | 5,173 | 4,656 | 4,352 | 4,512 | 4,750 | 6,189 | 4,749 |
| RVA/Human-wt/THA/NP-130/2014/G8P[8] | 1,299,694 | Nucleotide; bp  (% coverage of the full-length) | 1062  (100%) | 2351  (99.7%) | 1355  (99.9%) | 3297  (99.8%) | 2673  (99.6%) | 2585  (99.8%) | 1566  (100%) | 1038  (98.0%) | 1066  (100%) | 724  (96.4%) | 791  (96.9%) |
|  |  | Deduced amino acids; aa  (% coverage of the full-length) | 326  (100%) | 775  (100%) | 397  (100%) | 1088  (100%) | 879  (100%) | 835  (100%) | 486  (100%) | 317  (100%) | 313  (100%) | 175  (100%) | 200  (100%) |
|  |  | Reads mapped to gene segment  (% on-target reads of the total reads) | 36,250  (2.8%) | 115,855  (8.9%) | 31,472  (2.4%) | 100,088  (7.7%) | 149,635  (11.5%) | 99,780  (7.7%) | 38,655  (3.0%) | 34,794  (2.7%) | 47,278  (3.6%) | 11,842  (0.9%) | 10,676  (0.8%) |
|  |  | Maximum depth of reads | 7,786 | 6,669 | 8,044 | 6,983 | 10,536 | 4,598 | 6,137 | 5,815 | 4,584 | 6,000 | 3,123 |
| RVA/Human-wt/THA/PCB-656/2014/G8P[8] | 1,270,678 | Nucleotide; bp  (% coverage of the full-length) | 1061  (99.9%) | 2359  (100%) | 1356  (100%) | 3291  (99.7%) | 2673  (99.6%) | 2590  (99.9%) | 1564  (99.9%) | 1048  (99.0%) | 1066  (100%) | 736  (98.0%) | 804  (98.5%) |
|  |  | Deduced amino acids; aa  (% coverage of the full-length) | 326  (100%) | 775  (100%) | 397  (100%) | 1088  (100%) | 879  (100%) | 835  (100%) | 486  (100%) | 317  (100%) | 313  (100%) | 175  (100%) | 200  (100%) |
|  |  | Reads mapped to gene segment  (% on-target reads of the total reads) | 33,389  (2.6%) | 106,360  (8.4%) | 28,907  (2.3%) | 91,795  (7.2%) | 133,506  (10.5%) | 93,913  (7.4%) | 33,860  (2.7%) | 29,307  (2.3%) | 44,125  (3.5%) | 10,016  (0.8%) | 9,775  (0.8%) |
|  |  | Maximum depth of reads | 7,535 | 6,020 | 6,597 | 6,042 | 9,076 | 4,291 | 6,074 | 5,038 | 4,544 | 4,011 | 2,861 |
| RVA/Human-wt/THA/SKT-457/2014/G8P[8] | 563,370 | Nucleotide; bp  (% coverage of the full-length) | 1059  (99.7%) | 2358  (99.9%) | 1318  (97.2%) | 3298  (99.9%) | 2670  (99.5%) | 2577  (99.5%) | 1558  (99.5%) | 1046  (98.8%) | 1061  (99.5%) | 725  (96.5%) | 754  (92.4%) |
|  |  | Deduced amino acids; aa  (% coverage of the full-length) | 326  (100%) | 775  (100%) | 397  (100%) | 1088  (100%) | 879  (100%) | 835  (100%) | 486  (100%) | 317  (100%) | 313  (100%) | 175  (100%) | 200  (100%) |
|  |  | Reads mapped to gene segment  (% on-target reads of the total reads) | 34,815  (6.2%) | 47,598  (8.4%) | 11,786  (2.1%) | 62,576  (11.1%) | 51,635  (9.2%) | 44,512  (7.9%) | 23,883  (4.2%) | 22,608  (4.0%) | 28,469  (5.1%) | 2,764  (0.5%) | 1,371  (0.2%) |
|  |  | Maximum depth of reads | 4,998 | 4,142 | 4,126 | 3,521 | 3,788 | 2,584 | 4,340 | 4,052 | 4,514 | 1,152 | 568 |
| RVA/Human-wt/THA/SSKT-269/2014/G8P[8] | 1,072,288 | Nucleotide; bp  (% coverage of the full-length) | 1062  (100%) | 2358  (99.9%) | 1352  (99.7%) | 3294  (99.8%) | 2674  (99.6%) | 2584  (99.7%) | 1566  (100%) | 1048  (99.0%) | 1066  (100%) | 729  (97.1%) | 765  (93.8%) |
|  |  | Deduced amino acids; aa  (% coverage of the full-length) | 326  (100%) | 775  (100%) | 397  (100%) | 1088  (100%) | 879  (100%) | 835  (100%) | 486  (100%) | 317  (100%) | 313  (100%) | 175  (100%) | 200  (100%) |
|  |  | Reads mapped to gene segment  (% on-target reads of the total reads) | 49,027  (4.6%) | 77,300  (7.2%) | 15,755  (1.5%) | 99,625  (9.3%) | 90,087  (8.4%) | 66,610  (6.2%) | 26,953  (2.5%) | 30,954  (2.9%) | 30,801  (2.9%) | 6,267  (0.6%) | 2,149  (0.2%) |
|  |  | Maximum depth of reads | 7,652 | 5,924 | 4,326 | 5,785 | 7,051 | 3,644 | 1,309 | 4,914 | 4,332 | 2,160 | 790 |
| RVA/Human-wt/THA/SSL-55/2014/G8P[8] | 837,814 | Nucleotide; bp  (% coverage of the full-length) | 1059  (99.7%) | 2359  (100%) | 1355  (99.9%) | 3296  (99.8%) | 2680  (99.9%) | 2590  (99.9%) | 1566  (100%) | 1050  (99.2%) | 1066  (100%) | 733  (97.6%) | 802  (98.3%) |
|  |  | Deduced amino acids; aa  (% coverage of the full-length) | 326  (100%) | 775  (100%) | 397  (100%) | 1088  (100%) | 879  (100%) | 835  (100%) | 486  (100%) | 317  (100%) | 313  (100%) | 175  (100%) | 200  (100%) |
|  |  | Reads mapped to gene segment  (% on-target reads of the total reads) | 61,447  (7.3%) | 87,332  (10.4%) | 18,788  (2.2%) | 127,660  (15.2%) | 94,898  (11.3%) | 78,733  (9.4%) | 32,648  (3.9%) | 38,445  (4.6%) | 37,648  (4.5%) | 7,364  (0.9%) | 4,621  (0.6%) |
|  |  | Maximum depth of reads | 9,500 | 7,166 | 5,867 | 6,471 | 8,630 | 4,147 | 6,613 | 6,193 | 5,185 | 2,571 | 1,288 |
| RVA/Human-wt/THA/LS-202/2014/G2P[4] | 615,412 | Nucleotide; bp  (% coverage of the full-length) | 1061  (99.9%) | 2351  (99.7%) | 1345  (99.2%) | 3300  (99.9%) | 2674  (99.6%) | 2577  (99.5%) | 1554  (99.2%) | 1047  (98.9%) | 1065  (99.9%) | 737  (98.1%) | 784  (96.1%) |
|  |  | Deduced amino acids; aa  (% coverage of the full-length) | 326  (100%) | 775  (100%) | 397  (100%) | 1088  (100%) | 879  (100%) | 835  (100%) | 486  (100%) | 317  (100%) | 313  (100%) | 175  (100%) | 200  (100%) |
|  |  | Reads mapped to gene segment  (% on-target reads of the total reads) | 22,837  (3.7%) | 42,557  (6.9%) | 11,829  (1.9%) | 77,777  (12.6%) | 62,006  (10.1%) | 48,198  (7.8%) | 22,173  (3.6%) | 24,339  (4.0%) | 42,557  (6.9%) | 5,413  (0.9%) | 2,022  (0.3%) |
|  |  | Maximum depth of reads | 3,403 | 3,626 | 3,567 | 3,454 | 4,352 | 2,720 | 3,623 | 3,730 | 3,625 | 2,312 | 700 |
| RVA/Human-wt/THA/LS-L7/2014/G2P[4] | 648,000 | Nucleotide; bp  (% coverage of the full-length) | 1060  (99.8%) | 2351  (99.7%) | 1353  (99.8%) | 3294  (99.8%) | 2674  (99.6%) | 2591  (100%) | 1564  (99.9%) | 1047  (98.9%) | 1065  (99.9%) | 725  (96.5%) | 816  (100%) |
|  |  | Deduced amino acids; aa  (% coverage of the full-length) | 326  (100%) | 775  (100%) | 397  (100%) | 1088  (100%) | 879  (100%) | 835  (100%) | 486  (100%) | 317  (100%) | 313  (100%) | 175  (100%) | 200  (100%) |
|  |  | Reads mapped to gene segment  (% on-target reads of the total reads) | 17,463  (2.7%) | 41,932  (6.5%) | 11,162  (1.7%) | 46,173  (7.1%) | 44,368  (6.8%) | 32,749  (5.1%) | 24,781  (3.8%) | 15,727  (2.4%) | 24,762  (3.8%) | 5,494  (0.8%) | 1,170  (0.2%) |
|  |  | Maximum depth of reads | 2,258 | 3,481 | 3,018 | 2,452 | 2,421 | 2,240 | 3,644 | 2,324 | 3,644 | 2,145 | 498 |

^a^Sequence reads remaining after adapter trimming.
