# Supplementary material for: Full Genome Characterization of Novel DS-1-Like G8P[8] Rotavirus Strains that Have Emerged in Thailand: Reassortment of Bovine and Human Rotavirus Gene Segments in Emerging DS-1-Like Intergenogroup Reassortant Strains
Source: PLoS One. 2016 Nov 1;11(11):e0165826. doi: 10.1371/journal.pone.0165826 (PMC5089778; doi:10.1371/journal.pone.0165826)
Supplement: S3 Table — (DOCX) [file pone.0165826.s003.docx]

**S3 Table.** Nucleotide sequence identities (%) of the 11 gene segments of 12 Thai DS-1-like G8P[8] strains, KKL-17, PCB-79, PCB-84, PCB-85, PCB-103, SKT-107, SWL-12, NP-130, PCB-656, SKT-457, SSKT-269, and SSL-55, to one another.

| Gene | Study strain (nucleotide sequence identity) | | | | | | | | | | | |
| --- | --- | --- | --- | --- | --- | --- | --- | --- | --- | --- | --- | --- |
|  | KKL-17 | PCB-79 | PCB-84 | PCB-85 | PCB-103 | SKT-107 | SWL-12 | NP-130 | PCB-656 | SKT-457 | SSKT-269 | SSL-55 |
| VP7 | PCB-79 (100%)  PCB-84 (99.9%)  PCB-85 (100%)  PCB-103 (100%)  SKT-107 (100%)  SWL-12 (100%)  NP-130 (99.8%)  PCB-656 (99.7%)  SKT-457 (99.6%)  SSKT-269 (99.7%)  SSL-55 (99.7%) | KKL-17 (100%)  PCB-84 (99.9%)  PCB-85 (100%)  PCB-103 (100%)  SKT-107 (100%)  SWL-12 (100%)  NP-130 (99.8%)  PCB-656 (99.7%)  SKT-457 (99.6%)  SSKT-269 (99.7%)  SSL-55 (99.7%) | KKL-17 (99.9%)  PCB-79 (99.9%)  PCB-85 (99.9%)  PCB-103 (99.9%)  SKT-107 (99.9%)  SWL-12 (99.9%)  NP-130 (99.7%)  PCB-656 (99.6%)  SKT-457 (99.5%)  SSKT-269 (99.6%)  SSL-55 (99.6%) | KKL-17 (100%)  PCB-79 (100%)  PCB-84 (99.9%)  PCB-103 (100%)  SKT-107 (100%)  SWL-12 (100%)  NP-130 (99.8%)  PCB-656 (99.7%)  SKT-457 (99.6%)  SSKT-269 (99.7%)  SSL-55 (99.7%) | KKL-17 (100%)  PCB-79 (100%)  PCB-84 (99.9%)  PCB-85 (100%)  SKT-107 (100%)  SWL-12 (100%)  NP-130 (99.8%)  PCB-656 (99.7%)  SKT-457 (99.6%)  SSKT-269 (99.7%)  SSL-55 (99.7%) | KKL-17 (100%)  PCB-79 (100%)  PCB-84 (99.9%)  PCB-85 (100%)  PCB-103 (100%)  SWL-12 (100%)  NP-130 (99.8%)  PCB-656 (99.7%)  SKT-457 (99.6%)  SSKT-269 (99.7%)  SSL-55 (99.7%) | KKL-17 (100%)  PCB-79 (100%)  PCB-84 (99.9%)  PCB-85 (100%)  PCB-103 (100%)  SKT-107 (100%)  NP-130 (99.8%)  PCB-656 (99.7%)  SKT-457 (99.6%)  SSKT-269 (99.7%)  SSL-55 (99.7%) | KKL-17 (99.8%)  PCB-79 (99.8%)  PCB-84 (99.7%)  PCB-85 (99.8%)  PCB-103 (99.8%)  SKT-107 (99.8%)  SWL-12 (99.8%)  PCB-656 (99.9%)  SKT-457 (99.8%)  SSKT-269 (99.9%)  SSL-55 (99.9%) | KKL-17 (99.7%)  PCB-79 (99.7%)  PCB-84 (99.6%)  PCB-85 (99.7%)  PCB-103 (99.7%)  SKT-107 (99.7%)  SWL-12 (99.7%)  NP-130 (99.9%)  SKT-457 (99.9%)  SSKT-269 (100%)  SSL-55 (100%) | KKL-17 (99.6%)  PCB-79 (99.6%)  PCB-84 (99.5%)  PCB-85 (99.6%)  PCB-103 (99.6%)  SKT-107 (99.6%)  SWL-12 (99.6%)  NP-130 (99.8%)  PCB-656 (99.9%)  SSKT-269 (99.9%)  SSL-55 (99.9%) | KKL-17 (99.7%)  PCB-79 (99.7%)  PCB-84 (99.6%)  PCB-85 (99.7%)  PCB-103 (99.7%)  SKT-107 (99.7%)  SWL-12 (99.7%)  NP-130 (99.9%)  PCB-656 (100%)  SKT-457 (99.9%)  SSL-55 (100%) | KKL-17 (99.7%)  PCB-79 (99.7%)  PCB-84 (99.6%)  PCB-85 (99.7%)  PCB-103 (99.7%)  SKT-107 (99.7%)  SWL-12 (99.7%)  NP-130 (99.9%)  PCB-656 (100%)  SKT-457 (99.9%)  SSKT-269 (100%) |
| VP4 | PCB-79 (100%)  PCB-84 (100%)  PCB-85 (99.9%)  PCB-103 (100%)  SKT-107 (100%)  SWL-12 (100%)  NP-130 (99.8%)  PCB-656 (99.7%)  SKT-457 (99.8%)  SSKT-269 (99.8%)  SSL-55 (99.8%) | KKL-17 (100%)  PCB-84 (100%)  PCB-85 (99.9%)  PCB-103 (100%)  SKT-107 (100%)  SWL-12 (100%)  NP-130 (99.8%)  PCB-656 (99.7%)  SKT-457 (99.8%)  SSKT-269 (99.8%)  SSL-55 (99.8%) | KKL-17 (100%)  PCB-79 (100%)  PCB-85 (99.9%)  PCB-103 (100%)  SKT-107 (100%)  SWL-12 (100%)  NP-130 (99.8%)  PCB-656 (99.7%)  SKT-457 (99.8%)  SSKT-269 (99.8%)  SSL-55 (99.8%) | KKL-17 (99.9%)  PCB-79 (99.9%)  PCB-84 (99.9%)  PCB-103 (99.9%)  SKT-107 (99.9%)  SWL-12 (99.9%)  NP-130 (99.8%)  PCB-656 (99.7%)  SKT-457 (99.8%)  SSKT-269 (99.8%)  SSL-55 (99.9%) | KKL-17 (100%)  PCB-79 (100%)  PCB-84 (100%)  PCB-85 (99.9%)  SKT-107 (100%)  SWL-12 (100%)  NP-130 (99.8%)  PCB-656 (99.7%)  SKT-457 (99.8%)  SSKT-269 (99.8%)  SSL-55 (99.8%) | KKL-17 (100%)  PCB-79 (100%)  PCB-84 (100%)  PCB-85 (99.9%)  PCB-103 (100%)  SWL-12 (100%)  NP-130 (99.8%)  PCB-656 (99.7%)  SKT-457 (99.8%)  SSKT-269 (99.8%)  SSL-55 (99.8%) | KKL-17 (100%)  PCB-79 (100%)  PCB-84 (100%)  PCB-85 (99.9%)  PCB-103 (100%)  SKT-107 (100%)  NP-130 (99.8%)  PCB-656 (99.7%)  SKT-457 (99.8%)  SSKT-269 (99.8%)  SSL-55 (99.8%) | KKL-17 (99.8%)  PCB-79 (99.8%)  PCB-84 (99.8%)  PCB-85 (99.8%)  PCB-103 (99.8%)  SKT-107 (99.8%)  SWL-12 (99.8%)  PCB-656 (99.7%)  SKT-457 (99.9%)  SSKT-269 (99.9%)  SSL-55 (99.9%) | KKL-17 (99.7%)  PCB-79 (99.7%)  PCB-84 (99.7%)  PCB-85 (99.7%)  PCB-103 (99.7%)  SKT-107 (99.7%)  SWL-12 (99.7%)  NP-130 (99.7%)  SKT-457 (99.7%)  SSKT-269 (99.7%)  SSL-55 (99.8%) | KKL-17 (99.8%)  PCB-79 (99.8%)  PCB-84 (99.8%)  PCB-85 (99.8%)  PCB-103 (99.8%)  SKT-107 (99.8%)  SWL-12 (99.8%)  NP-130 (99.9%)  PCB-656 (99.7%)  SSKT-269 (100%)  SSL-55 (99.9%) | KKL-17 (99.8%)  PCB-79 (99.8%)  PCB-84 (99.8%)  PCB-85 (99.8%)  PCB-103 (99.8%)  SKT-107 (99.8%)  SWL-12 (99.8%)  NP-130 (99.9%)  PCB-656 (99.7%)  SKT-457 (100%)  SSL-55 (99.9%) | KKL-17 (99.8%)  PCB-79 (99.8%)  PCB-84 (99.8%)  PCB-85 (99.9%)  PCB-103 (99.8%)  SKT-107 (99.8%)  SWL-12 (99.8%)  NP-130 (99.9%)  PCB-656 (99.8%)  SKT-457 (99.9%)  SSKT-269 (99.9%) |
| VP6 | PCB-79 (100%)  PCB-84 (100%)  PCB-85 (99.9%)  PCB-103 (100%)  SKT-107 (100%)  SWL-12 (100%)  NP-130 (92.0%)  PCB-656 (91.9%)  SKT-457 (91.8%)  SSKT-269 (92.0%)  SSL-55 (91.9%) | KKL-17 (100%)  PCB-84 (100%)  PCB-85 (99.9%)  PCB-103 (100%)  SKT-107 (100%)  SWL-12 (100%)  NP-130 (92.0%)  PCB-656 (91.9%)  SKT-457 (91.8%)  SSKT-269 (92.0%)  SSL-55 (91.9%) | KKL-17 (100%)  PCB-79 (100%)  PCB-85 (99.9%)  PCB-103 (100%)  SKT-107 (100%)  SWL-12 (100%)  NP-130 (92.0%)  PCB-656 (91.9%)  SKT-457 (91.8%)  SSKT-269 (92.0%)  SSL-55 (91.9%) | KKL-17 (99.9%)  PCB-79 (99.9%)  PCB-84 (99.9%)  PCB-103 (99.9%)  SKT-107 (99.9%)  SWL-12 (99.9%)  NP-130 (92.0%)  PCB-656 (91.9%)  SKT-457 (91.8%)  SSKT-269 (91.9%)  SSL-55 (91.9%) | KKL-17 (100%)  PCB-79 (100%)  PCB-84 (100%)  PCB-85 (99.9%)  SKT-107 (100%)  SWL-12 (100%)  NP-130 (92.0%)  PCB-656 (91.9%)  SKT-457 (91.8%)  SSKT-269 (92.0%)  SSL-55 (91.9%) | KKL-17 (100%)  PCB-79 (100%)  PCB-84 (100%)  PCB-85 (99.9%)  PCB-103 (100%)  SWL-12 (100%)  NP-130 (92.0%)  PCB-656 (91.9%)  SKT-457 (91.8%)  SSKT-269 (92.0%)  SSL-55 (91.9%) | KKL-17 (100%)  PCB-79 (100%)  PCB-84 (100%)  PCB-85 (99.9%)  PCB-103 (100%)  SKT-107 (100%)  NP-130 (92.0%)  PCB-656 (91.9%)  SKT-457 (91.8%)  SSKT-269 (92.0%)  SSL-55 (91.9%) | KKL-17 (92.0%)  PCB-79 (92.0%)  PCB-84 (92.0%)  PCB-85 (92.0%)  PCB-103 (92.0%)  SKT-107 (92.0%)  SWL-12 (92.0%)  PCB-656 (99.9%)  SKT-457 (99.7%)  SSKT-269 (100%)  SSL-55 (99.9%) | KKL-17 (91.9%)  PCB-79 (91.9%)  PCB-84 (91.9%)  PCB-85 (91.9%)  PCB-103 (91.9%)  SKT-107 (91.9%)  SWL-12 (91.9%)  NP-130 (99.9%)  SKT-457 (99.6%)  SSKT-269 (99.9%)  SSL-55 (99.8%) | KKL-17 (91.8%)  PCB-79 (91.8%)  PCB-84 (91.8%)  PCB-85 (91.8%)  PCB-103 (91.8%)  SKT-107 (91.8%)  SWL-12 (91.8%)  NP-130 (99.7%)  PCB-656 (99.6%)  SSKT-269 (99.7%)  SSL-55 (99.6%) | KKL-17 (92.0%)  PCB-79 (92.0%)  PCB-84 (92.0%)  PCB-85 (92.0%)  PCB-103 (92.0%)  SKT-107 (92.0%)  SWL-12 (92.0%)  NP-130 (100%)  PCB-656 (99.7%)  SKT-457 (99.7%)  SSL-55 (99.9%) | KKL-17 (91.9%)  PCB-79 (91.9%)  PCB-84 (91.9%)  PCB-85 (91.9%)  PCB-103 (91.9%)  SKT-107 (91.9%)  SWL-12 (91.9%)  NP-130 (99.9%)  PCB-656 (99.8%)  SKT-457 (99.6%)  SSKT-269 (99.9%) |
| VP1 | PCB-79 (100%)  PCB-84 (100%)  PCB-85 (99.9%)  PCB-103 (100%)  SKT-107 (100%)  SWL-12 (100%)  NP-130 (99.9%)  PCB-656 (99.8%)  SKT-457 (99.9%)  SSKT-269 (99.9%)  SSL-55 (99.8%) | KKL-17 (100%)  PCB-84 (100%)  PCB-85 (99.9%)  PCB-103 (100%)  SKT-107 (100%)  SWL-12 (100%)  NP-130 (99.9%)  PCB-656 (99.8%)  SKT-457 (99.9%)  SSKT-269 (99.9%)  SSL-55 (99.8%) | KKL-17 (100%)  PCB-79 (100%)  PCB-85 (99.9%)  PCB-103 (100%)  SKT-107 (100%)  SWL-12 (100%)  NP-130 (99.9%)  PCB-656 (99.8%)  SKT-457 (99.9%)  SSKT-269 (99.9%)  SSL-55 (99.8%) | KKL-17 (99.9%)  PCB-79 (99.9%)  PCB-84 (99.9%)  PCB-103 (99.9%)  SKT-107 (99.9%)  SWL-12 (99.9%)  NP-130 (99.9%)  PCB-656 (99.8%)  SKT-457 (99.9%)  SSKT-269 (99.9%)  SSL-55 (99.8%) | KKL-17 (100%)  PCB-79 (100%)  PCB-84 (100%)  PCB-85 (99.9%)  SKT-107 (100%)  SWL-12 (100%)  NP-130 (99.9%)  PCB-656 (99.8%)  SKT-457 (99.9%)  SSKT-269 (99.9%)  SSL-55 (99.8%) | KKL-17 (100%)  PCB-79 (100%)  PCB-84 (100%)  PCB-85 (99.9%)  PCB-103 (100%)  SWL-12 (100%)  NP-130 (99.9%)  PCB-656 (99.8%)  SKT-457 (99.9%)  SSKT-269 (99.9%)  SSL-55 (99.8%) | KKL-17 (100%)  PCB-79 (100%)  PCB-84 (100%)  PCB-85 (99.9%)  PCB-103 (100%)  SKT-107 (100%)  NP-130 (99.9%)  PCB-656 (99.8%)  SKT-457 (99.9%)  SSKT-269 (99.9%)  SSL-55 (99.8%) | KKL-17 (99.9%)  PCB-79 (99.9%)  PCB-84 (99.9%)  PCB-85 (99.9%)  PCB-103 (99.9%)  SKT-107 (99.9%)  SWL-12 (99.9%)  PCB-656 (99.9%)  SKT-457 (99.9%)  SSKT-269 (99.9%)  SSL-55 (99.9%) | KKL-17 (99.8%)  PCB-79 (99.8%)  PCB-84 (99.8%)  PCB-85 (99.8%)  PCB-103 (99.8%)  SKT-107 (99.8%)  SWL-12 (99.8%)  NP-130 (99.9%)  SKT-457 (99.9%)  SSKT-269 (99.9%)  SSL-55 (99.9%) | KKL-17 (99.9%)  PCB-79 (99.9%)  PCB-84 (99.9%)  PCB-85 (99.9%)  PCB-103 (99.9%)  SKT-107 (99.9%)  SWL-12 (99.9%)  NP-130 (99.9%)  PCB-656 (99.9%)  SSKT-269 (100%)  SSL-55 (99.9%) | KKL-17 (99.9%)  PCB-79 (99.9%)  PCB-84 (99.9%)  PCB-85 (99.9%)  PCB-103 (99.9%)  SKT-107 (99.9%)  SWL-12 (99.9%)  NP-130 (99.9%)  PCB-656 (99.9%)  SKT-457 (100%)  SSL-55 (99.9%) | KKL-17 (99.8%)  PCB-79 (99.8%)  PCB-84 (99.8%)  PCB-85 (99.8%)  PCB-103 (99.8%)  SKT-107 (99.8%)  SWL-12 (99.8%)  NP-130 (99.9%)  PCB-656 (99.9%)  SKT-457 (99.9%)  SSKT-269 (99.9%) |
| VP2 | PCB-79 (100%)  PCB-84 (100%)  PCB-85 (100%)  PCB-103 (100%)  SKT-107 (100%)  SWL-12 (100%)  NP-130 (99.9%)  PCB-656 (99.9%)  SKT-457 (99.8%)  SSKT-269 (99.8%)  SSL-55 (99.9%) | KKL-17 (100%)  PCB-84 (100%)  PCB-85 (100%)  PCB-103 (100%)  SKT-107 (100%)  SWL-12 (100%)  NP-130 (99.9%)  PCB-656 (99.9%)  SKT-457 (99.8%)  SSKT-269 (99.8%)  SSL-55 (99.9%) | KKL-17 (100%)  PCB-79 (100%)  PCB-85 (100%)  PCB-103 (100%)  SKT-107 (100%)  SWL-12 (100%)  NP-130 (99.9%)  PCB-656 (99.9%)  SKT-457 (99.8%)  SSKT-269 (99.8%)  SSL-55 (99.9%) | KKL-17 (100%)  PCB-79 (100%)  PCB-84 (100%)  PCB-103 (100%)  SKT-107 (100%)  SWL-12 (100%)  NP-130 (99.9%)  PCB-656 (99.9%)  SKT-457 (99.8%)  SSKT-269 (99.8%)  SSL-55 (99.9%) | KKL-17 (100%)  PCB-79 (100%)  PCB-84 (100%)  PCB-85 (100%)  SKT-107 (100%)  SWL-12 (100%)  NP-130 (99.9%)  PCB-656 (99.9%)  SKT-457 (99.8%)  SSKT-269 (99.8%)  SSL-55 (99.9%) | KKL-17 (100%)  PCB-79 (100%)  PCB-84 (100%)  PCB-85 (100%)  PCB-103 (100%)  SWL-12 (100%)  NP-130 (99.9%)  PCB-656 (99.9%)  SKT-457 (99.8%)  SSKT-269 (99.8%)  SSL-55 (99.9%) | KKL-17 (100%)  PCB-79 (100%)  PCB-84 (100%)  PCB-85 (100%)  PCB-103 (100%)  SKT-107 (100%)  NP-130 (99.9%)  PCB-656 (99.9%)  SKT-457 (99.8%)  SSKT-269 (99.8%)  SSL-55 (99.9%) | KKL-17 (99.9%)  PCB-79 (99.9%)  PCB-84 (99.9%)  PCB-85 (99.9%)  PCB-103 (99.9%)  SKT-107 (99.9%)  SWL-12 (99.9%)  PCB-656 (99.8%)  SKT-457 (99.8%)  SSKT-269 (99.7%)  SSL-55 (99.8%) | KKL-17 (99.9%)  PCB-79 (99.9%)  PCB-84 (99.9%)  PCB-85 (99.9%)  PCB-103 (99.9%)  SKT-107 (99.9%)  SWL-12 (99.9%)  NP-130 (99.8%)  SKT-457 (99.9%)  SSKT-269 (99.8%)  SSL-55 (99.9%) | KKL-17 (99.8%)  PCB-79 (99.8%)  PCB-84 (99.8%)  PCB-85 (99.8%)  PCB-103 (99.8%)  SKT-107 (99.8%)  SWL-12 (99.8%)  NP-130 (99.8%)  PCB-656 (99.9%)  SSKT-269 (99.9%)  SSL-55 (99.8%) | KKL-17 (99.8%)  PCB-79 (99.8%)  PCB-84 (99.8%)  PCB-85 (99.8%)  PCB-103 (99.8%)  SKT-107 (99.8%)  SWL-12 (99.8%)  NP-130 (99.7%)  PCB-656 (99.8%)  SKT-457 (99.9%)  SSL-55 (99.8%) | KKL-17 (99.9%)  PCB-79 (99.9%)  PCB-84 (99.9%)  PCB-85 (99.9%)  PCB-103 (99.9%)  SKT-107 (99.9%)  SWL-12 (99.9%)  NP-130 (99.8%)  PCB-656 (99.9%)  SKT-457 (99.8%)  SSKT-269 (99.8%) |
| VP3 | PCB-79 (100%)  PCB-84 (99.9%)  PCB-85 (99.9%)  PCB-103 (99.9%)  SKT-107 (100%)  SWL-12 (100%)  NP-130 (99.8%)  PCB-656 (99.9%)  SKT-457 (99.8%)  SSKT-269 (99.8%)  SSL-55 (99.8%) | KKL-17 (100%)  PCB-84 (99.9%)  PCB-85 (99.9%)  PCB-103 (99.9%)  SKT-107 (100%)  SWL-12 (100%)  NP-130 (99.8%)  PCB-656 (99.9%)  SKT-457 (99.8%)  SSKT-269 (99.8%)  SSL-55 (99.8%) | KKL-17 (99.9%)  PCB-79 (99.9%)  PCB-85 (100%)  PCB-103 (100%)  SKT-107 (99.9%)  SWL-12 (99.9%)  NP-130 (99.9%)  PCB-656 (100%)  SKT-457 (99.9%)  SSKT-269 (99.9%)  SSL-55 (99.8%) | KKL-17 (99.9%)  PCB-79 (99.9%)  PCB-84 (100%)  PCB-103 (100%)  SKT-107 (99.9%)  SWL-12 (99.9%)  NP-130 (99.9%)  PCB-656 (100%)  SKT-457 (99.9%)  SSKT-269 (99.9%)  SSL-55 (99.8%) | KKL-17 (99.9%)  PCB-79 (99.9%)  PCB-84 (100%)  PCB-85 (100%)  SKT-107 (99.9%)  SWL-12 (99.9%)  NP-130 (99.9%)  PCB-656 (100%)  SKT-457 (99.9%)  SSKT-269 (99.9%)  SSL-55 (99.8%) | KKL-17 (100%)  PCB-79 (100%)  PCB-84 (99.9%)  PCB-85 (99.9%)  PCB-103 (99.9%)  SWL-12 (100%)  NP-130 (99.8%)  PCB-656 (99.9%)  SKT-457 (99.8%)  SSKT-269 (99.8%)  SSL-55 (99.8%) | KKL-17 (100%)  PCB-79 (100%)  PCB-84 (99.9%)  PCB-85 (99.9%)  PCB-103 (99.9%)  SKT-107 (100%)  NP-130 (99.8%)  PCB-656 (99.9%)  SKT-457 (99.8%)  SSKT-269 (99.8%)  SSL-55 (99.8%) | KKL-17 (99.8%)  PCB-79 (99.8%)  PCB-84 (99.9%)  PCB-85 (99.9%)  PCB-103 (99.9%)  SKT-107 (99.8%)  SWL-12 (99.8%)  PCB-656 (99.8%)  SKT-457 (99.9%)  SSKT-269 (99.9%)  SSL-55 (99.8%) | KKL-17 (99.9%)  PCB-79 (99.9%)  PCB-84 (99.8%)  PCB-85 (99.8%)  PCB-103 (99.8%)  SKT-107 (99.9%)  SWL-12 (99.9%)  NP-130 (99.8%)  SKT-457 (99.8%)  SSKT-269 (99.8%)  SSL-55 (99.8%) | KKL-17 (99.8%)  PCB-79 (99.8%)  PCB-84 (99.9%)  PCB-85 (99.9%)  PCB-103 (99.9%)  SKT-107 (99.8%)  SWL-12 (99.8%)  NP-130 (99.9%)  PCB-656 (99.8%)  SSKT-269 (100%)  SSL-55 (99.8%) | KKL-17 (99.8%)  PCB-79 (99.8%)  PCB-84 (99.9%)  PCB-85 (99.9%)  PCB-103 (99.9%)  SKT-107 (99.8%)  SWL-12 (99.8%)  NP-130 (99.9%)  PCB-656 (99.8%)  SKT-457 (100%)  SSL-55 (99.8%) | KKL-17 (99.8%)  PCB-79 (99.8%)  PCB-84 (99.8%)  PCB-85 (99.8%)  PCB-103 (99.8%)  SKT-107 (99.8%)  SWL-12 (99.8%)  NP-130 (99.8%)  PCB-656 (99.8%)  SKT-457 (99.8%)  SSKT-269 (99.8%) |
| NSP1 | PCB-79 (100%)  PCB-84 (100%)  PCB-85 (100%)  PCB-103 (100%)  SKT-107 (100%)  SWL-12 (100%)  NP-130 (99.9%)  PCB-656 (99.8%)  SKT-457 (99.8%)  SSKT-269 (99.8%)  SSL-55 (99.8%) | KKL-17 (100%)  PCB-84 (100%)  PCB-85 (100%)  PCB-103 (100%)  SKT-107 (100%)  SWL-12 (100%)  NP-130 (99.9%)  PCB-656 (99.8%)  SKT-457 (99.8%)  SSKT-269 (99.8%)  SSL-55 (99.8%) | KKL-17 (100%)  PCB-79 (100%)  PCB-85 (100%)  PCB-103 (100%)  SKT-107 (100%)  SWL-12 (100%)  NP-130 (99.9%)  PCB-656 (99.8%)  SKT-457 (99.8%)  SSKT-269 (99.8%)  SSL-55 (99.8%) | KKL-17 (100%)  PCB-79 (100%)  PCB-84 (100%)  PCB-103 (100%)  SKT-107 (100%)  SWL-12 (100%)  NP-130 (99.9%)  PCB-656 (99.8%)  SKT-457 (99.8%)  SSKT-269 (99.8%)  SSL-55 (99.8%) | KKL-17 (100%)  PCB-79 (100%)  PCB-84 (100%)  PCB-85 (100%)  SKT-107 (100%)  SWL-12 (100%)  NP-130 (99.9%)  PCB-656 (99.8%)  SKT-457 (99.8%)  SSKT-269 (99.8%)  SSL-55 (99.8%) | KKL-17 (100%)  PCB-79 (100%)  PCB-84 (100%)  PCB-85 (100%)  PCB-103 (100%)  SWL-12 (100%)  NP-130 (99.9%)  PCB-656 (99.8%)  SKT-457 (99.8%)  SSKT-269 (99.8%)  SSL-55 (99.8%) | KKL-17 (100%)  PCB-79 (100%)  PCB-84 (100%)  PCB-85 (100%)  PCB-103 (100%)  SKT-107 (100%)  NP-130 (99.9%)  PCB-656 (99.8%)  SKT-457 (99.8%)  SSKT-269 (99.8%)  SSL-55 (99.8%) | KKL-17 (99.9%)  PCB-79 (99.9%)  PCB-84 (99.9%)  PCB-85 (99.9%)  PCB-103 (99.9%)  SKT-107 (99.9%)  SWL-12 (99.9%)  PCB-656 (99.8%)  SKT-457 (99.8%)  SSKT-269 (99.8%)  SSL-55 (99.8%) | KKL-17 (99.8%)  PCB-79 (99.8%)  PCB-84 (99.8%)  PCB-85 (99.8%)  PCB-103 (99.8%)  SKT-107 (99.8%)  SWL-12 (99.8%)  NP-130 (99.8%)  SKT-457 (99.8%)  SSKT-269 (99.8%)  SSL-55 (99.8%) | KKL-17 (99.8%)  PCB-79 (99.8%)  PCB-84 (99.8%)  PCB-85 (99.8%)  PCB-103 (99.8%)  SKT-107 (99.8%)  SWL-12 (99.8%)  NP-130 (99.8%)  PCB-656 (99.8%)  SSKT-269 (100%)  SSL-55 (99.8%) | KKL-17 (99.8%)  PCB-79 (99.8%)  PCB-84 (99.8%)  PCB-85 (99.8%)  PCB-103 (99.8%)  SKT-107 (99.8%)  SWL-12 (99.8%)  NP-130 (99.8%)  PCB-656 (99.8%)  SKT-457 (100%)  SSL-55 (99.8%) | KKL-17 (99.8%)  PCB-79 (99.8%)  PCB-84 (99.8%)  PCB-85 (99.8%)  PCB-103 (99.8%)  SKT-107 (99.8%)  SWL-12 (99.8%)  NP-130 (99.8%)  PCB-656 (99.8%)  SKT-457 (99.8%)  SSKT-269 (99.8%) |
| NSP2 | PCB-79 (100%)  PCB-84 (99.9%)  PCB-85 (99.8%)  PCB-103 (99.9%)  SKT-107 (100%)  SWL-12 (100%)  NP-130 (99.7%)  PCB-656 (99.7%)  SKT-457 (99.7%)  SSKT-269 (99.7%)  SSL-55 (99.8%) | KKL-17 (100%)  PCB-84 (99.9%)  PCB-85 (99.8%)  PCB-103 (99.9%)  SKT-107 (100%)  SWL-12 (100%)  NP-130 (99.7%)  PCB-656 (99.7%)  SKT-457 (99.7%)  SSKT-269 (99.7%)  SSL-55 (99.8%) | KKL-17 (99.9%)  PCB-79 (99.9%)  PCB-85 (99.9%)  PCB-103 (100%)  SKT-107 (99.9%)  SWL-12 (99.9%)  NP-130 (99.8%)  PCB-656 (99.8%)  SKT-457 (99.8%)  SSKT-269 (99.8%)  SSL-55 (99.9%) | KKL-17 (99.8%)  PCB-79 (99.8%)  PCB-84 (99.9%)  PCB-103 (99.9%)  SKT-107 (99.8%)  SWL-12 (99.8%)  NP-130 (99.9%)  PCB-656 (99.9%)  SKT-457 (99.9%)  SSKT-269 (99.9%)  SSL-55 (100%) | KKL-17 (99.9%)  PCB-79 (99.9%)  PCB-84 (100%)  PCB-85 (99.9%)  SKT-107 (99.9%)  SWL-12 (99.9%)  NP-130 (99.8%)  PCB-656 (99.8%)  SKT-457 (99.8%)  SSKT-269 (99.8%)  SSL-55 (99.9%) | KKL-17 (100%)  PCB-79 (100%)  PCB-84 (99.9%)  PCB-85 (99.8%)  PCB-103 (99.9%)  SWL-12 (100%)  NP-130 (99.7%)  PCB-656 (99.7%)  SKT-457 (99.7%)  SSKT-269 (99.7%)  SSL-55 (99.8%) | KKL-17 (100%)  PCB-79 (100%)  PCB-84 (99.9%)  PCB-85 (99.8%)  PCB-103 (99.9%)  SKT-107 (100%)  NP-130 (99.7%)  PCB-656 (99.7%)  SKT-457 (99.7%)  SSKT-269 (99.7%)  SSL-55 (99.8%) | KKL-17 (99.7%)  PCB-79 (99.7%)  PCB-84 (99.8%)  PCB-85 (99.9%)  PCB-103 (99.8%)  SKT-107 (99.7%)  SWL-12 (99.7%)  PCB-656 (99.8%)  SKT-457 (99.8%)  SSKT-269 (99.8%)  SSL-55 (99.9%) | KKL-17 (99.7%)  PCB-79 (99.7%)  PCB-84 (99.8%)  PCB-85 (99.9%)  PCB-103 (99.8%)  SKT-107 (99.7%)  SWL-12 (99.7%)  NP-130 (99.8%)  SKT-457 (99.8%)  SSKT-269 (99.8%)  SSL-55 (99.9%) | KKL-17 (99.7%)  PCB-79 (99.7%)  PCB-84 (99.8%)  PCB-85 (99.9%)  PCB-103 (99.8%)  SKT-107 (99.7%)  SWL-12 (99.7%)  NP-130 (99.8%)  PCB-656 (99.8%)  SSKT-269 (100%)  SSL-55 (99.9%) | KKL-17 (99.7%)  PCB-79 (99.7%)  PCB-84 (99.8%)  PCB-85 (99.9%)  PCB-103 (99.8%)  SKT-107 (99.7%)  SWL-12 (99.7%)  NP-130 (99.8%)  PCB-656 (99.8%)  SKT-457 (100%)  SSL-55 (99.9%) | KKL-17 (99.8%)  PCB-79 (99.8%)  PCB-84 (99.9%)  PCB-85 (100%)  PCB-103 (99.9%)  SKT-107 (99.8%)  SWL-12 (99.8%)  NP-130 (99.9%)  PCB-656 (99.9%)  SKT-457 (99.9%)  SSKT-269 (99.9%) |
| NSP3 | PCB-79 (100%)  PCB-84 (100%)  PCB-85 (100%)  PCB-103 (99.9%)  SKT-107 (100%)  SWL-12 (100%)  NP-130 (99.8%)  PCB-656 (100%)  SKT-457 (99.9%)  SSKT-269 (100%)  SSL-55 (99.9%) | KKL-17 (100%)  PCB-84 (100%)  PCB-85 (100%)  PCB-103 (99.9%)  SKT-107 (100%)  SWL-12 (100%)  NP-130 (99.8%)  PCB-656 (100%)  SKT-457 (99.9%)  SSKT-269 (100%)  SSL-55 (99.9%) | KKL-17 (100%)  PCB-79 (100%)  PCB-85 (100%)  PCB-103 (99.9%)  SKT-107 (100%)  SWL-12 (100%)  NP-130 (99.8%)  PCB-656 (100%)  SKT-457 (99.9%)  SSKT-269 (100%)  SSL-55 (99.9%) | KKL-17 (100%)  PCB-79 (100%)  PCB-84 (100%)  PCB-103 (99.9%)  SKT-107 (100%)  SWL-12 (100%)  NP-130 (99.8%)  PCB-656 (100%)  SKT-457 (99.9%)  SSKT-269 (100%)  SSL-55 (99.9%) | KKL-17 (99.9%)  PCB-79 (99.9%)  PCB-84 (99.9%)  PCB-85 (99.9%)  SKT-107 (99.9%)  SWL-12 (99.9%)  NP-130 (99.7%)  PCB-656 (99.9%)  SKT-457 (99.8%)  SSKT-269 (99.9%)  SSL-55 (99.9%) | KKL-17 (100%)  PCB-79 (100%)  PCB-84 (100%)  PCB-85 (100%)  PCB-103 (99.9%)  SWL-12 (100%)  NP-130 (99.8%)  PCB-656 (100%)  SKT-457 (99.9%)  SSKT-269 (100%)  SSL-55 (99.9%) | KKL-17 (100%)  PCB-79 (100%)  PCB-84 (100%)  PCB-85 (100%)  PCB-103 (99.9%)  SKT-107 (100%)  NP-130 (99.8%)  PCB-656 (100%)  SKT-457 (99.9%)  SSKT-269 (100%)  SSL-55 (99.9%) | KKL-17 (99.8%)  PCB-79 (99.8%)  PCB-84 (99.8%)  PCB-85 (99.8%)  PCB-103 (99.7%)  SKT-107 (99.8%)  SWL-12 (99.8%)  PCB-656 (99.8%)  SKT-457 (99.8%)  SSKT-269 (99.8%)  SSL-55 (99.7%) | KKL-17 (100%)  PCB-79 (100%)  PCB-84 (100%)  PCB-85 (100%)  PCB-103 (99.9%)  SKT-107 (100%)  SWL-12 (100%)  NP-130 (99.8%)  SKT-457 (99.9%)  SSKT-269 (100%)  SSL-55 (99.9%) | KKL-17 (99.9%)  PCB-79 (99.9%)  PCB-84 (99.9%)  PCB-85 (99.9%)  PCB-103 (99.8%)  SKT-107 (99.9%)  SWL-12 (99.9%)  NP-130 (99.8%)  PCB-656 (99.9%)  SSKT-269 (99.9%)  SSL-55 (99.8%) | KKL-17 (100%)  PCB-79 (100%)  PCB-84 (100%)  PCB-85 (100%)  PCB-103 (99.9%)  SKT-107 (100%)  SWL-12 (100%)  NP-130 (99.8%)  PCB-656 (100%)  SKT-457 (99.9%)  SSL-55 (99.9%) | KKL-17 (99.9%)  PCB-79 (99.9%)  PCB-84 (99.9%)  PCB-85 (99.9%)  PCB-103 (99.8%)  SKT-107 (99.9%)  SWL-12 (99.9%)  NP-130 (99.7%)  PCB-656 (99.9%)  SKT-457 (99.8%)  SSKT-269 (99.9%) |
| NSP4 | PCB-79 (100%)  PCB-84 (99.8%)  PCB-85 (100%)  PCB-103 (99.8%)  SKT-107 (100%)  SWL-12 (100%)  NP-130 (99.7%)  PCB-656 (99.5%)  SKT-457 (99.5%)  SSKT-269 (99.7%)  SSL-55 (99.7%) | KKL-17 (100%)  PCB-84 (99.8%)  PCB-85 (100%)  PCB-103 (99.8%)  SKT-107 (100%)  SWL-12 (100%)  NP-130 (99.7%)  PCB-656 (99.5%)  SKT-457 (99.5%)  SSKT-269 (99.7%)  SSL-55 (99.7%) | KKL-17 (99.8%)  PCB-79 (99.8%)  PCB-85 (99.8%)  PCB-103 (100%)  SKT-107 (99.8%)  SWL-12 (99.8%)  NP-130 (99.5%)  PCB-656 (99.4%)  SKT-457 (99.4%)  SSKT-269 (99.5%)  SSL-55 (99.5%) | KKL-17 (100%)  PCB-79 (100%)  PCB-84 (99.8%)  PCB-103 (99.8%)  SKT-107 (100%)  SWL-12 (100%)  NP-130 (99.7%)  PCB-656 (99.5%)  SKT-457 (99.5%)  SSKT-269 (99.7%)  SSL-55 (99.7%) | KKL-17 (99.8%)  PCB-79 (99.8%)  PCB-84 (100%)  PCB-85 (99.8%)  SKT-107 (99.8%)  SWL-12 (99.8%)  NP-130 (99.5%)  PCB-656 (99.4%)  SKT-457 (99.4%)  SSKT-269 (99.5%)  SSL-55 (99.5%) | KKL-17 (100%)  PCB-79 (100%)  PCB-84 (99.8%)  PCB-85 (100%)  PCB-103 (99.8%)  SWL-12 (100%)  NP-130 (99.7%)  PCB-656 (99.5%)  SKT-457 (99.5%)  SSKT-269 (99.7%)  SSL-55 (99.7%) | KKL-17 (100%)  PCB-79 (100%)  PCB-84 (99.8%)  PCB-85 (100%)  PCB-103 (99.8%)  SKT-107 (100%)  NP-130 (99.7%)  PCB-656 (99.5%)  SKT-457 (99.5%)  SSKT-269 (99.7%)  SSL-55 (99.7%) | KKL-17 (99.7%)  PCB-79 (99.7%)  PCB-84 (99.5%)  PCB-85 (99.7%)  SKT-103 (99.5%)  SKT-107 (99.7%)  SWL-12 (99.7%)  PCB-656 (99.5%)  SKT-457 (99.5%)  SSKT-269 (99.7%)  SSL-55 (99.7%) | KKL-17 (99.5%)  PCB-79 (99.5%)  PCB-84 (99.4%)  PCB-85 (99.5%)  PCB-103 (99.4%)  SKT-107 (99.5%)  SWL-12 (99.5%)  NP-130 (99.5%)  SKT-457 (99.7%)  SSKT-269 (99.8%)  SSL-55 (99.8%) | KKL-17 (99.5%)  PCB-79 (99.5%)  PCB-84 (99.4%)  PCB-85 (99.5%)  PCB-103 (99.4%)  SKT-107 (99.5%)  SWL-12 (99.5%)  NP-130 (99.5%)  PCB-656 (99.7%)  SSKT-269 (99.8%)  SSL-55 (99.8%) | KKL-17 (99.7%)  PCB-79 (99.7%)  PCB-84 (99.5%)  PCB-85 (99.7%)  PCB-103 (99.5%)  SKT-107 (99.7%)  SWL-12 (99.7%)  NP-130 (99.7%)  PCB-656 (99.8%)  SKT-457 (99.8%)  SSL-55 (100%) | KKL-17 (99.7%)  PCB-79 (99.7%)  PCB-84 (99.5%)  PCB-85 (99.7%)  PCB-103 (99.5%)  SKT-107 (99.7%)  SWL-12 (99.7%)  NP-130 (99.7%)  PCB-656 (99.8%)  SKT-457 (99.8%)  SSKT-269 (100%) |
| NSP5 | PCB-79 (100%)  PCB-84 (100%)  PCB-85 (100%)  PCB-103 (100%)  SKT-107 (100%)  SWL-12 (99.8%)  NP-130 (98.9%)  PCB-656 (99.3%)  SKT-457 (96.3%)  SSKT-269 (96.4%)  SSL-55 (99.3%) | KKL-17 (100%)  PCB-84 (100%)  PCB-85 (100%)  PCB-103 (100%)  SKT-107 (100%)  SWL-12 (99.8%)  NP-130 (98.9%)  PCB-656 (99.3%)  SKT-457 (96.3%)  SSKT-269 (96.4%)  SSL-55 (99.3%) | KKL-17 (100%)  PCB-79 (100%)  PCB-85 (100%)  PCB-103 (100%)  SKT-107 (100%)  SWL-12 (99.8%)  NP-130 (98.9%)  PCB-656 (99.3%)  SKT-457 (96.3%)  SSKT-269 (96.4%)  SSL-55 (99.3%) | KKL-17 (100%)  PCB-79 (100%)  PCB-84 (100%)  PCB-103 (100%)  SKT-107 (100%)  SWL-12 (99.8%)  NP-130 (98.9%)  PCB-656 (99.3%)  SKT-457 (96.3%)  SSKT-269 (96.4%)  SSL-55 (99.3%) | KKL-17 (100%)  PCB-79 (100%)  PCB-84 (100%)  PCB-85 (100%)  SKT-107 (100%)  SWL-12 (99.8%)  NP-130 (98.9%)  PCB-656 (99.3%)  SKT-457 (96.3%)  SSKT-269 (96.4%)  SSL-55 (99.3%) | KKL-17 (100%)  PCB-79 (100%)  PCB-84 (100%)  PCB-85 (100%)  PCB-103 (100%)  SWL-12 (99.8%)  NP-130 (98.9%)  PCB-656 (99.3%)  SKT-457 (96.3%)  SSKT-269 (96.4%)  SSL-55 (99.3%) | KKL-17 (99.8%)  PCB-79 (99.8%)  PCB-84 (99.8%)  PCB-85 (99.8%)  PCB-103 (99.8%)  SKT-107 (99.8%)  NP-130 (98.8%)  PCB-656 (99.2%)  SKT-457 (96.2%)  SSKT-269 (96.2%)  SSL-55 (99.2%) | KKL-17 (98.9%)  PCB-79 (98.9%)  PCB-84 (98.9%)  PCB-85 (98.9%)  PCB-103 (98.9%)  SKT-107 (98.9%)  SWL-12 (98.8%)  PCB-656 (99.6%)  SKT-457 (95.8%)  SSKT-269 (95.9%)  SSL-55 (99.6%) | KKL-17 (99.3%)  PCB-79 (99.3%)  PCB-84 (99.3%)  PCB-85 (99.3%)  PCB-103 (99.3%)  SKT-107 (99.3%)  SWL-12 (99.2%)  NP-130 (99.6%)  SKT-457 (96.2%)  SSKT-269 (96.2%)  SSL-55 (100%) | KKL-17 (96.3%)  PCB-79 (96.3%)  PCB-84 (96.3%)  PCB-85 (96.3%)  PCB-103 (96.3%)  SKT-107 (96.3%)  SWL-12 (96.2%)  NP-130 (95.8%)  PCB-656 (96.2%)  SSKT-269 (100%)  SSL-55 (96.2%) | KKL-17 (96.3%)  PCB-79 (96.3%)  PCB-84 (96.3%)  PCB-85 (96.3%)  PCB-103 (96.3%)  SKT-107 (96.3%)  SWL-12 (96.2%)  NP-130 (95.8%)  PCB-656 (96.2%)  SKT-457 (100%)  SSL-55 (96.2%) | KKL-17 (99.3%)  PCB-79 (99.3%)  PCB-84 (99.3%)  PCB-85 (99.3%)  PCB-103 (99.3%)  SKT-107 (99.3%)  SWL-12 (99.2%)  NP-130 (99.6%)  PCB-656 (96.2%)  SKT-457 (96.2%)  SSKT-269 (100%) |
